# Primary care services role in supporting families of children or adolescents with disabilities or neurodevelopmental disorders*

**DOI:** 10.1590/1980-220X-REEUSP-2025-0152en

**Published:** 2026-02-16

**Authors:** Luciano Silveira Pacheco de Medeiros, Thiago Ferreira Abreu, Viviane Marten Milbrath, Ruth Irmgard Bärtschi Gabatz, Sônia Silva Marcon, Luciano Garcia Lourenção, Giovana Calcagno Gomes, Clarice Alves Bonow

**Affiliations:** 1 Universidade Federal de Pelotas, Faculdade de Enfermagem, Programa de Pós-Graduação em Enfermagem, Pelotas, RS, Brazil.; 2 Universidade Estadual de Maringá, Programa de Pós-Graduação em Enfermagem, Maringá, PR, Brazil.; 3 Universidade Federal do Rio Grande, Escola de Enfermagem, Programa de Pós-Graduação em Enfermagem, Rio Grande, RS, Brazil.

**Keywords:** Nursing Care, Children with Disability, Neurodevelopmental Disorders, Family, Primary Health Care

## Abstract

**Objective:**

To map and synthesize the care practices, strategies, and interventions developed in Primary Health Care for families of children and adolescents with disabilities and/or neurodevelopmental disorders.

**Method:**

Scoping review conducted according to the Joanna Briggs Institute guidelines and the PRISMA-ScR checklist. The following databases were consulted: LILACS, IBECS, Scielo, CINAHL, WoS, MEDLINE/PubMed, Scopus, and PsycINFO, including studies published between 2015 and 2024, in Portuguese, English, and Spanish.

**Results:**

Thirty-one studies were included, allowing for the construction of two thematic categories: experienced feelings and family dynamics; and aspects that weaken the performance of primary health care. Difficulties in access, a prescriptive care model, limited listening to families, and insufficient technical training of professionals were evident.

**Conclusion:**

Primary Health Care still has significant gaps in providing comprehensive and equitable care to families of children with disabilities and/or neurodevelopmental disorders. Overcoming these challenges requires skilled listening, intersectoral coordination, and recognition of these families as the central focus of care.

## INTRODUCTION

The modern society lives longer and with better quality of life. Among the benefits of modernity, we can cite all the technologies assisting humanity in its daily tasks, thus providing more time for activities other than those necessary for survival. However, all these available facilities contribute significantly to the increase in physical inactivity, the number of people with chronic non-communicable diseases (NCDs), acquired physical disabilities and/or associated comorbidities, among others^(1)^. People are living longer, but often in unhealthy conditions, resulting in individuals dependent on medium- and long-term care, frequent hospitalizations, and even preventable and premature deaths^(1)^.

The increase in life expectancy experienced globally, coupled with technological discoveries and innovations in healthcare, contributes to the growth in the number of people with these conditions. Studies conducted in the United States, for example, indicate that 18.5% of children and adolescents aged between zero and 17 years have some disability, neurodevelopmental disorder, or special health care needs – a group also known by the Portuguese acronym *CRIANES* – and will require complex and continuous long-term health care. This will involve several hospitalizations, the use of assistive technologies to provide better quality of life, and also monitoring by Primary Health Care professionals^(2,3)^.

In Brazil, although updated epidemiological data on this population is lacking, the Brazilian Institute of Geography and Statistics (IBGE) identified approximately 45.6 million people with disabilities in the 2010 Census, of which about 760,000 are children and/or adolescents^(2,3)^. Although such data lack systematic updates, they are indicative of a scenario of great relevance for the formulation of public policies and care practices.

The World Health Organization (WHO) defines disability as a broad term encompassing long-term physical, mental, intellectual, or sensory impairments which, in interaction with various barriers, may hinder full and effective participation in society on an equal basis with others. Neurodevelopmental disorders, on the other hand, refer to conditions that manifest early and affect the personal, social, or academic functioning of children, requiring multidisciplinary and continuous care. The Brazilian Law for the Inclusion of Persons with Disabilities (LBI - Law No. 13.146/2015), aligned with the UN Convention on the Rights of Persons with Disabilities (Decree No. 6.949/2009), reaffirms the right to health, social participation, and dignity of these populations, calling on health services to provide equitable, comprehensive, and accessible care.

The Convention on the Rights of Persons with Disabilities, approved by the United Nations in 2006, was incorporated into the Brazilian legal system with constitutional equivalence, pursuant to paragraph 3 of article 5 of the Federal Constitution. Together with the Brazilian Inclusion Law (Law No. 13.146/2015), it constitutes the main legal framework for guaranteeing the right to comprehensive health and social participation for people with disabilities.

Not long ago, for each role to be properly fulfilled within society—the man being the provider and the woman the caregiver—the birth of different children was not accepted^(4)^. Children with disabilities or chronic illnesses, as well as the older people, are no longer as frequently left behind for the sake of the survival of the rest of the group as they once were. However, the experience of motherhood/fatherhood of children with disabilities or neurodevelopmental disorders is still an experience that tends to negatively impact family dynamics, with crises frequently arising^(5)^. Family dynamics change, new health demands arise, situations of ableism and exclusion become commonplace, and support networks tend to weaken, which can lead to feelings of grief for the living child—that is, the feeling of loss that parents tend to experience even in the presence of their child^(5,6,7,8)^.

It is important to highlight that, often, health services still operate under a biomedical and pathologizing logic, centered on the disease and not on the person, which reinforces the exclusion of children with disabilities and makes the leading role of their families invisible. This body-normative perspective has to be critically analyzed so that progress can be made in building more equitable, intersectoral, and person-centered care practices.

In this scenario, Primary Health Care (PHC), being the main entry point to the Brazilian Public Health System (*SUS*) and closest to the daily lives of families, should be the first space for embracement, longitudinal care, and construction of relationships with these populations. According to the Pan American Health Organization (PAHO), PHC is responsible for identifying and responding, in a continuous and timely manner, to the needs of individuals and communities, prioritizing person-centered actions based on principles of equity, comprehensiveness, and social justice.

Based on the above, this study aimed to map and synthesize the care practices, strategies, and interventions developed in Primary Health Care for families of children and adolescents with disabilities and/or neurodevelopmental disorders.

## METHOD

### Design of Study

This is a scoping review^(9)^, conducted in accordance with the methodological guidelines of the *Joanna Briggs Institute - Manual for Evidence Synthesis and Preferred Reporting Items for Systematic reviews and Meta-Analyses extension for Scoping Reviews (PRISMA-ScR) Checklist*. Scoping reviews aim to map and synthesize current evidence available in the literature, highlighting knowledge gaps regarding a particular phenomenon and encouraging further research^(10)^. The protocol for this review is registered on the platform *Open Science Framework* (OSF) with DOI no. https://doi.org/10.17605/OSF.IO/Q4NSZ.

The proposed methodological steps were followed in its execution, namely: a) identification of the research question, b) identification of relevant studies, c) selection of studies, d) data mapping, and e) collection, summarization, and reporting of results^(10)^. Considering the search strategy used based on the acronym PCC (Population, Concept, Context), the following guiding research question was developed: “What are the care practices, strategies, and interventions available in national and international literature, developed in PHC, aimed at families of children and adolescents with disabilities and/or neurodevelopmental disorders?”, where P (Population): Families of children and adolescents with disabilities and/or neurodevelopmental disorders; C (Concept): Practices, strategies, and interventions that characterize caregiving; and, C (Context): PHC.

### Data Collection

For information extraction, publications indexed in the data-bases of Latin American and Caribbean Literature in Health Sciences (LILACS) and the Spanish Bibliographic Index of Health Sciences (IBECS), *Scientific Electronic Library Online* (Scielo), *Current Index to Nursing and Allied Health Literature* (CINAHL), *Web of Science* (WoS), *Medical Literature Analysis and Retrieval System Online* (MEDLINE/Pubmed) *SciVerse Scopus* (Scopus) *American Psychological Association* (PsycINFO) were consulted during the months of January and February 2025.

The inclusion criteria were original studies made available in full, resulting from qualitative, quantitative, or mixed-methods studies, in Portuguese, English, and Spanish.

The linguistic delimitation to Portuguese, English, and Spanish is justified by the fact that they are the most widely used languages in the selected databases and correspond to the languages mastered by the research team. Furthermore, these languages account for the majority of relevant scientific output in the health field in the Americas, as demonstrated in previous systematic reviews.

The following were considered exclusion criteria: works published before 2015 and duplicate works. The time frame starting from 2015 is explained by the enactment of the Brazilian Law for the Inclusion of Persons with Disabilities (LBI – Law No. 13.146/2015)^(11)^, which established fundamental normative, ethical, and organizational guidelines for consolidating the rights of people with disabilities in Brazil. This legislation directly influenced health practices and policies within the scope of Primary Care.

### Search Strategy

The same search strategy was used in all databases consulted, with the aid of the Boolean operators AND and OR, using the Health Sciences Descriptors (DeCS) and *Medical Subject Headings* (MeSH): ((Family OR Caregivers) AND (“disabled children” OR adolescent OR “Neurodevelopmental Disorders” OR “Persons with Mental Disabilities”) AND “Primary Health Care” AND (“Nursing Care” OR “Nurses, Public Health” OR Nursing)).

This review chose not to include grey literature among the search sources, prioritizing studies available in indexed databases with more homogeneous and standardized selection criteria. This choice aimed to ensure greater traceability, methodological comparability, and reproducibility of results. It is recognized, however, that non-indexed literature may contain relevant contributions to the field and that its exclusion constitutes a limitation to be considered in the discussion of the findings.

The selection of studies took place in three stages: reading of the titles, reading of the abstracts, and reading of the full articles. The process was carried out simultaneously by two independent authors, ensuring blinding. There were no disagreements at any stage.

For the separation, summarization, and reporting of the results, a structured instrument was used that allowed the extraction of the following data: title, authors, year of publication, study design, objective, participants, local, proposing institution, publication journal, and main results.

### Data Analysis and Treatment

For data analysis and processing, a descriptive synthesis was initially carried out, identifying the main characteristics of the selected studies. Subsequently, a second synthesis, of a categorical nature, was carried out, in which the main findings of these studies were highlighted. In this step, the following was used: *R Interface for Multidimensional Analysis of Texts and Questionnaires* (IRAMUTEQ), version 0.8 alpha 7, using the lexical proximity technique, that is, by grouping similar texts into sections, with the consequent identification of common themes, using Descending Hierarchical Classification (DHC), also known as Reinert’s method^(12)^. The categories that emerged after using the software were discussed through the lens of Critical Discourse Analysis (CDA)^(13)^.

The analysis of the textual data was carried out using the IRAMUTEQ software, through Classification. Although the dendrogram is a relevant visual tool in this technique, the originally generated graphical representation could not be recovered, which is why it was not included in this manuscript. The information extracted through DHC, however, was described textually and guided the categorization of the findings.

### Ethical Aspects

Since this was a scoping review study, there was no need to submit the proposal to and have it reviewed by a Research Ethics Committee (*CEP*).

## RESULTS

In the identification process, 1,207 primary studies were located in the consulted databases: 216 (17.9%) in Scopus, 118 (9.8%) in WoS, 465 (38.5%) in MEDLINE/Pubmed, 13 (1.1%) in IBECS, 90 (7.4%) in LILACS, 79 (6.5%) in CINAHAL, 159 (13.2%) in PsycINFO, and 67 (5.6%) through searches in the citations and references of the works excluded in this stage - review articles.

**Figure 1:**
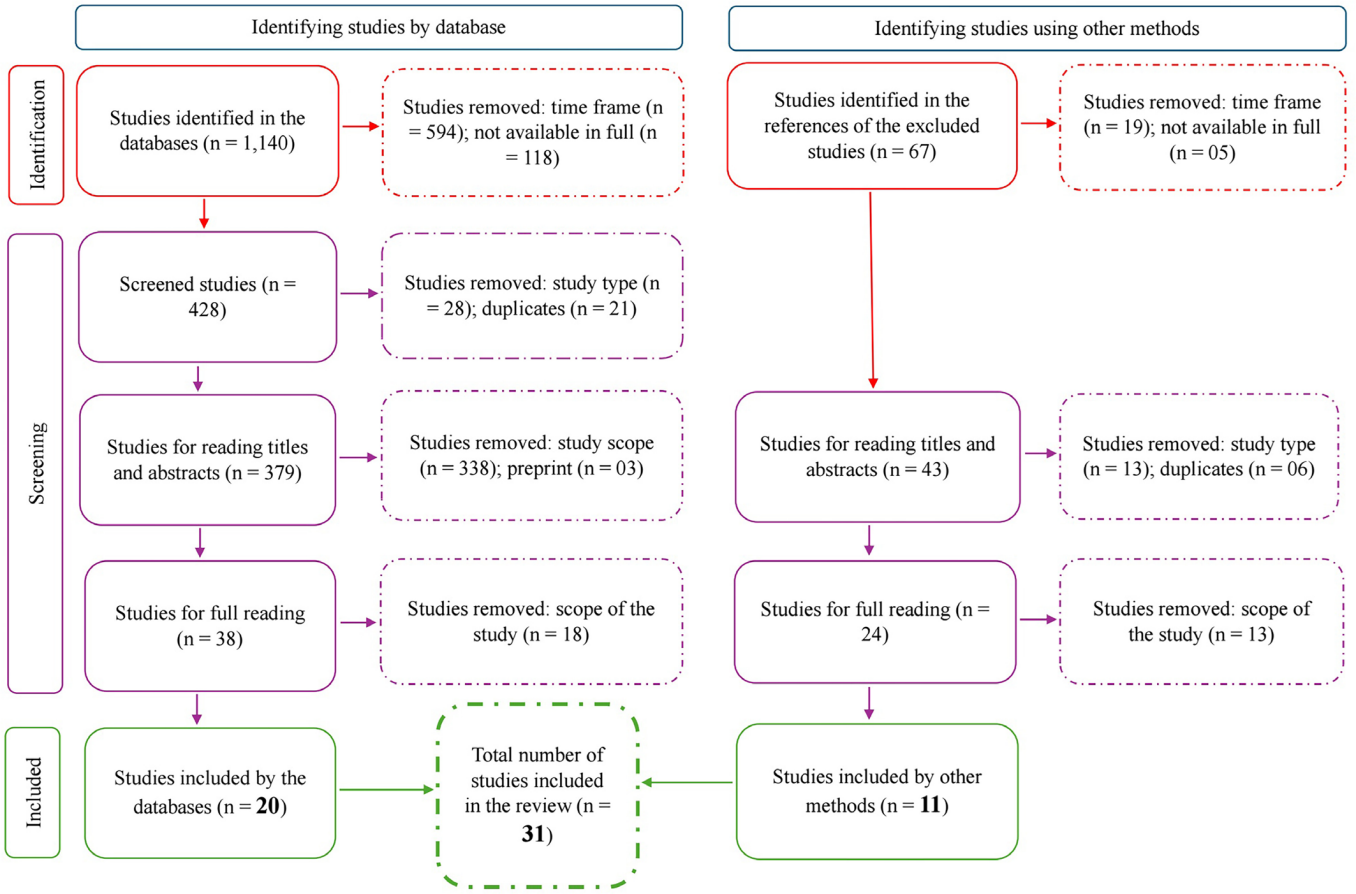
Eligibility process for studies found, according to PRISMA recommendations^(10)^ – Pelotas, RS, 2025.

Figure 1 illustrates the processes and steps followed for the selection of the studies found, according to PRISMA recommendations^(10)^.

**Figure 2:**
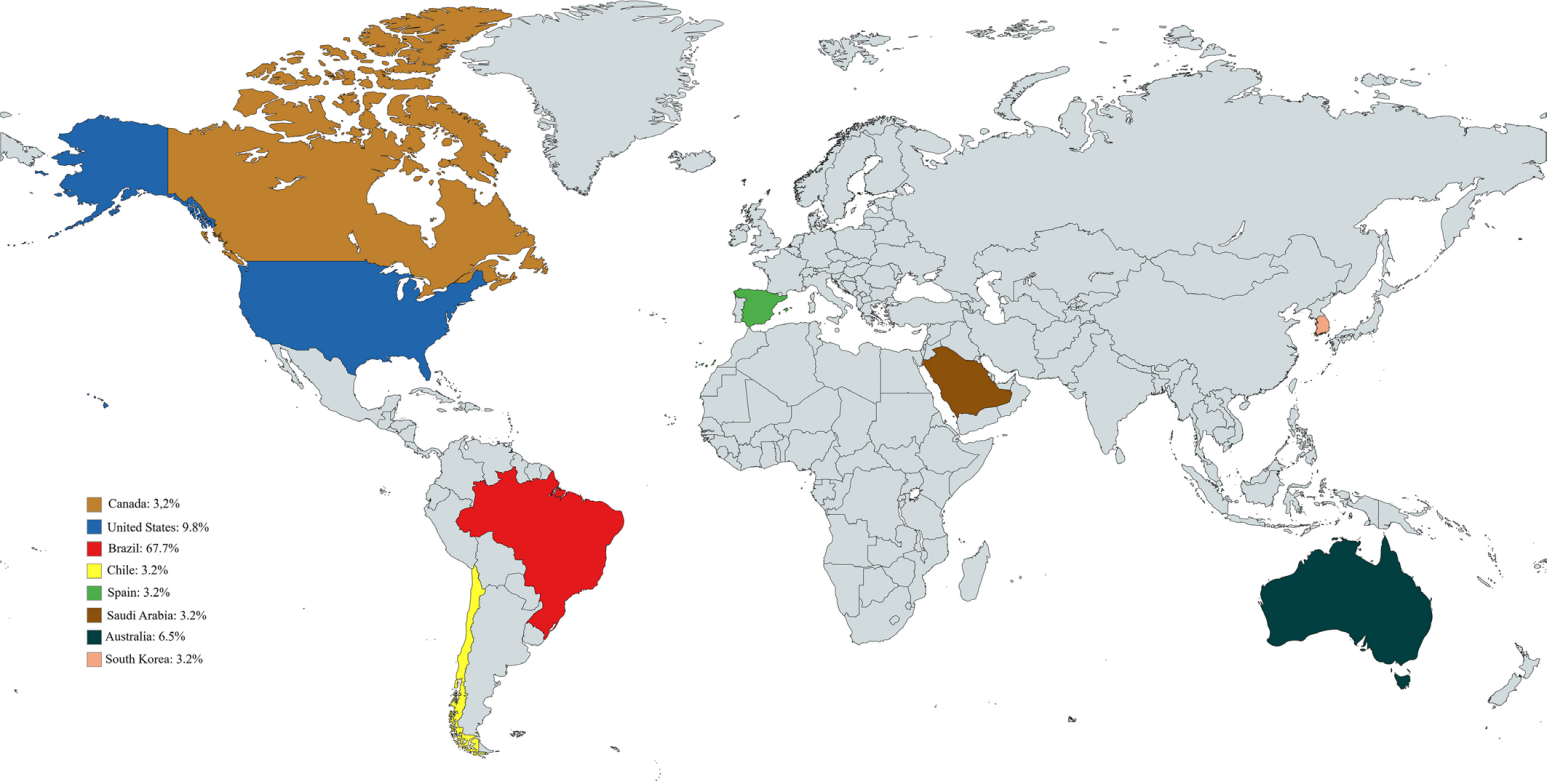
Spatial distribution, by country, of the selected studies – Pelotas, RS, 2025.

In the screening phase, 1,176 studies were excluded: 613 (52.1%) studies based on the year criterion, 123 (10.4%) on the availability criterion, 41 (3.5%) because they were review studies and manuals, 27 (2.3%) because they were duplicates, 369 (31.4%) because they were outside the proposed scope, and three (0.3%) because they were preprints, with 31 studies being eligible for discussion. Regarding the approach used in the studies, we have 24 (77.4%) qualitative studies^(14,15,16,17,18,19,20,21,22,23,24,25,26,27,28,29,30,31,32)^, six (19.4%) quantitative^(33,34,35,36,37,38)^ and one (3.2%) mixed methods study^(39)^. Of these, 18 (58.1%) studies were published in national journals.^(14,15,17,18,20,21,22,23,24,25,26,33,35,40,41,42,43,44)^ and 13 (41.9%) in international journals^(20,28,29,30,31,32,33,34,36,37,38,39,45)^, of various nationalities. Regarding their countries of origin, 21 (67.7%) were published in Brazil^(14,15,16,17,18,19,20,21,22,23,24,25,26,33,34,35,40,44)^, one (3.2%) in Chile^(36)^, one (3.2%) in Canada^(28)^, three (9.8%) in the United States^(29,30,37)^, one (3.2%) in Spain^(38)^, one (3.2%) in South Korea^(31)^, one (3.2%) in Saudi Arabia^(39)^, and two (6.5%) in Australia^(32,45)^, as shown in Figure 2.

Of the 31 studies included in this review, seven addressed exclusively conditions associated with disability (such as cerebral palsy, physical or intellectual disability), while 11 focused only on neurodevelopmental disorders (NDDs), such as autism spectrum disorder and attention deficit hyperactivity disorder (ADHD). Ten other studies simultaneously considered aspects of disability and NDD, highlighting the conceptual and clinical overlap between these conditions. Three studies did not provide sufficient information for precise categorization, as they dealt with cross-cutting themes or broader approaches. This distribution allows us to identify trends in scientific production and reinforces the importance of conceptually differentiating the groups analyzed, since family demands and the modes of care developed in PHC vary according to the predominant diagnosis.

Studies focusing exclusively on disability showed a predominance of practices centered on the functional rehabilitation of the child, with an emphasis on specialized referrals, the use of assistive devices, and specific guidance aimed at physical care and dependence on assistive technologies. In these cases, primary health care is frequently used as an entry point or referral point for obtaining benefits and material resources. Conversely, studies dealing only with neurodevelopmental disorders, such as autism spectrum disorder, placed greater emphasis on actions aimed at providing psychosocial support to families, coordinating with mental health and education services, and ensuring continuity of care. In studies considering both conditions, the findings show more integrated practices, although still marked by gaps in longitudinal follow-up and in listening to families. The analysis suggests that the predominant type of condition influences the focus of actions developed in primary health care, which demands technical preparation and sensitivity to recognize the complexity of the family contexts involved.

Most of the included studies focus on preschool and school-aged children, predominantly those between zero and twelve years old. There was a scarcity of studies that specifically address adolescents with disabilities or neurodevelopmental disorders in the context of Primary Health Care. Although some articles include adolescents in their samples, few develop specific analyses of the particularities of this phase of life. This age gap is mentioned by some authors as a methodological limitation, especially given the need to ensure continuity of care and strategies adapted to the transition process from childhood to adolescence. The lack of a specific focus on adolescents points to a significant healthcare challenge and the need to broaden the perspective of primary healthcare regarding this population.

Most of the studies analyzed refer to the Family Health Strategy (FHS) model, which is predominant in Brazil, describing the activities carried out by nurses, doctors, and community health workers. Studies were also identified that mention the role of conventional Primary Health Units and, to a lesser extent, primary care services organized in an integrated way within the school environment or linked to specific local policies, as observed in experiences in Chile, Spain, and Australia. Despite the diversity of arrangements, few studies thoroughly explore how the organizational model of primary health care influences the quality and continuity of care provided to families of children with disabilities or neurodevelopmental disorders. This analytical absence represents a gap in the literature and reinforces the importance of considering institutional contexts when evaluating healthcare practices.

Although the studies have been conducted in different regions of Brazil and in other countries, most do not describe the regional specificities of the contexts analyzed extensively, which constitutes a limitation for understanding the local dynamics of organization and access to primary care services.

**Figure 3:**
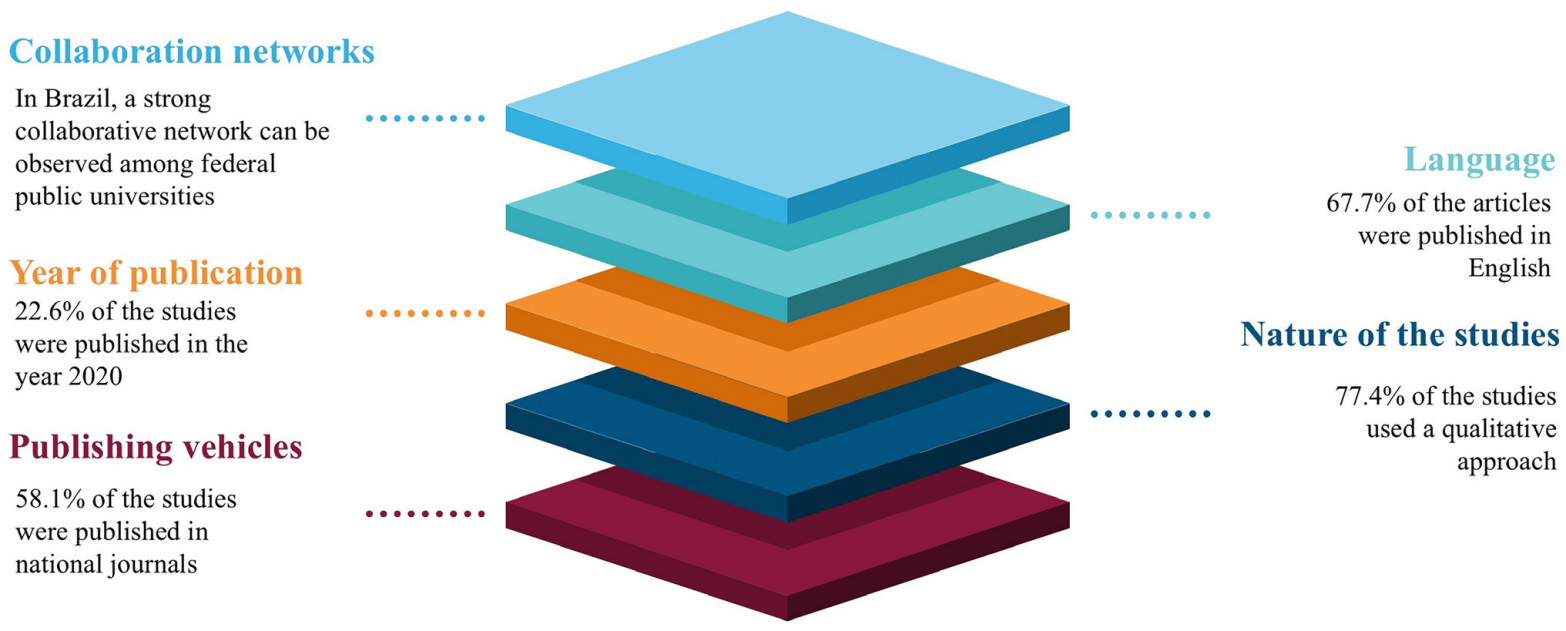
Main characteristics of the selected studies – Pelotas, RS, 2025.

Regarding language, articles published in English predominate (21–67.7%)^(16,17,18,19,20,26,28,29,30,31,32,34,36,37,38,39,41,44)^, nine (29.1%) are in Portuguese^(15,16,17,21,22,23,35,41,43,44)^ and one (3.2%) is in Spanish^(36)^. Regarding the year of publication, one (3.3%) article was published in 2015^(29)^, three (9.7%) in 2017^(15,16,33)^, four (12.9%) in 2018^(17,28,31,32)^, four (12.9%) in 2019^(18,19,20,34)^, seven (22.6%) in 2020^(21,35,40,41,42,43,44)^, four (12.9%) in 2021^(22,23,37,39)^, two (6.4%) in 2022^(24,45)^, two (6.4%) in 2023^(25,36)^ and four (12.9%) in 2024^(26,27,30,38)^ (Figure 3).

A strong collaborative network exists among faculty researchers from graduate programs at federal public universities, primarily in the South-Southeast-Central-West region^(18,19,21,22,23,24,25,26,27,33,35,41,42,43,44)^. This can be justified by the fact that Brazil has a unique, public, and free healthcare system with national coverage, capable of providing a minimally satisfactory response to the health demands presented by users, and which aims to serve as the entry point to PHC and resolve users’ demands, whenever possible, within their territory of origin^(46,47)^.

In addition to this, we have a network of federal public higher education institutions with excellent undergraduate and graduate programs, with diverse teaching, research, and extension activities, aimed at training and qualifying professionals capable of providing comprehensive and humanized care to the individual-family dyad^(48)^.

To facilitate the discussion of the results observed in the selected studies, the points were grouped into a synoptic chart: feelings experienced and family dynamics; and aspects that weaken PHC performance (Chart 1).

**Chart 1:** Social determinants of health and aspects that influence care in Primary Health Care (PHC) – Pelotas, RS, Brazil, 2025.

Experienced feelings and family dynamics	
Observed dimensions	Description of the findings
Negative feelings upon receiving the diagnosis.	‘And now?’. ‘What am I going to do?’ ‘Why me?’ ‘How am I going to take care of it?’ ‘Will my husband accept?’ ‘How am I going to pay for the treatments?’ are just some of the questions that come to families’ minds when a child is diagnosed with a disability or a possible neurodevelopmental disorder; families feel unprepared to face the situation currently presented^(18,19,27,34,36,41,43)^. Anxiety, fear, sadness, and grief over the loss of a living child are some of the feelings perceived, negatively impacting all dimensions of family dynamics, consequently decreasing the sense of well-being and leading to the onset and worsening of minor mental disorders^(15,21,22,23,28,31,32,33,37,38,39)^.
The process of invisibilization and exclusion of children and adolescents with neurodevelopmental disorders or disabilities and their families.	People with disabilities or neurodevelopmental disorders have faced a variety of challenges throughout history. Currently, they suffer from constant repression in their processes of subjectivation, resulting in their invisibility and alienation, as well as that of their families^(23,25,34,41)^. Children and adolescents with neurodevelopmental disorders or disabilities are considered to have less efficient bodies, containing less essence than bodies considered normative, and suffer from exclusionary and ableist practices that are relativized by strongly defended neoliberal policies. The materiality of their bodies can no longer be hidden, yet they remain marginalized by society, being exposed to prejudice, violence, digital influence, substance abuse, and crime^(15,16,18,22,28,29,30,31,32,33,36,38,39,43,44)^.
Predominance of women as the primary caregivers in the family.	The predominance of women as primary caregivers in the family and the perception of non-mutual parenting, leading to a decrease in reported well-being and quality of life, weaknesses in their significant social networks, or even their existence. This directly impacts the onset and/or worsening of minor mental health disorders such as anxiety, stress, and depression, which tends to lead to separations and divorces, further exacerbating already altered family dynamics^(17,19,22,25,28,32,37,40,43,45)^.
Lack of knowledge or preparedness on the part of the family regarding how to use medications, equipment, and assistive technology devices.	The regular handling and administration of medications, and the use of assistive equipment and technologies, generally accompany the diagnosis of a neurodevelopmental disability or disorder^(21,22,24,25,35,41,43,44)^. These families, mostly headed by mothers, constantly report a lack of knowledge and quite high levels of fear and unpreparedness to carry out activities now necessary to maintain their children’s health. This increases their dependence on long-term support and assistance from minimally qualified professionals, represented, in the minds of these families, by nursing professionals^(15,16,17,19,21,22,24,25,30,31,33,35,38,40,43,44)^.
Dependence on social security and welfare benefits	The socioeconomic and cultural contexts presented by these families are, to a large extent, complex and a result of low parental education levels combined with income, employment, housing, and access to healthcare services. This negatively impacts their living conditions, leading to an increasing need for social assistance and pension benefits, and an almost complete dependence on public education and health policies^(17,18,19,20,33,34,40,44)^.
Accessibility difficulties	Studies highlight the various accessibility barriers in Primary Health Care perceived by families, among which we can mention structural barriers, specific to the buildings of the health units responsible for the families assigned to the territories, and attitudinal barriers, represented by stigmatization, prejudice, and ableism perceived by families during and in order for care to take place^(15,16,18,19,21,23,24,25,33,34,36,40,41,44,45)^. These families also report constant trips between primary healthcare networks, underutilization, low problem-solving capacity, and the need to supplement their services by contracting private health insurance plans^(15,16,18,19,21,22,23,30,34,39,40,41,44)^.
**Aspects that weaken the performance of Primary Health Care**	
**Observed dimensions**	**Description of the findings**
Creating and strengthening bonds with families.	The creation and strengthening of bonds between nursing professionals and the families they care for is considered an important and powerful too^(19,20,27,29,36,40,41)^. This allows for the [re]cognition of the reality in which these families are embedded, their assigned territories, and their social determinants of health in a broader and more contextualized way; however, weak bonds are observed between nursing professionals and the families under their care, a fact that leads to sporadic care and a superficial understanding of the families’ lived reality^(17,18,21,22,24,26,30,32,33,35,37,42,43,44)^.
Underutilization of home visits and individualized therapeutic plans, and low effectiveness of health education initiatives.	It was observed that educational initiatives for promoting health practices were underutilized, especially those developed during home visits. The educational actions provided by nursing staff proved to be limited to teaching the use of materials, equipment, and medications^(15,17,18,19,20,33,35,40,43)^, not considering the family as an open and closed system in constant interaction with the environment, which allows it to exchange information and resources necessary for its needs^(49,50,51)^. The need for nursing care stems from a shift in care from a biomedical model to a social model of disability, also considering neurodevelopmental disorders, with the family as the ‘focus’ and a longitudinal approach to care^(22,23,24,26,28,29,30,31,32,37,39)^.
Coordinated work with specialized levels of medium and high complexity and other collaborative networks.	The healthcare needs presented are largely concentrated in the services and technologies available at the Primary Health Care Unit (UBS) assigned to their area of responsibility; however, in many situations, it is necessary to refer these families to specialized medium and high complexity levels of care^(17,21,25,26,27,28,36,41)^ and the need for constant and horizontal dialogue with Social Assistance, Education, and other collaborative networks identified as important for families within their assigned territory, with the determination of care pathways, service flows, therapeutic itineraries, the development of the Individual Therapeutic Project (ITP), and the strengthening of the referral and counter-referral system (CRS)^(15,16,18,23,34,39,45)^.

The first category addresses all the social determinants of health and their direct implications for the health needs of families with children and adolescents with neurodevelopmental disorders or disabilities: negative feelings upon receiving the diagnosis; the process of invisibility and exclusion of children and adolescents with neurodevelopmental disorders or disabilities and their families; the predominance of women as the primary caregivers in the family; the family’s lack of knowledge or preparedness in how to use medications, equipment, and assistive technology devices; dependence on social security and welfare benefits; and accessibility difficulties.

The second category discusses aspects related to the quantity and quality of care in PHC for families of children and adolescents with neurodevelopmental disorders or disabilities: creation and strengthening of bonds with families; underutilization of home visits and low effectiveness of health education actions; coordinated work with specialized levels of medium and high complexity and others in collaborative networks.

## DISCUSSION

The following discussion revisits the elements organized in Chart 1, which summarizes the main social determinants of health and aspects that influence care in PHC for families of children and adolescents with disabilities and/or neurodevelopmental disorders. The analysis was structured around two interpretative axes derived from the synthesis of the findings: a) Experienced feelings and family dynamics; and, b) Aspects that weaken PHC performance.

### Experienced Feelings and Family Dynamics

The diagnosis of a disability or neurodevelopmental disorder in a child and/or adolescent can present itself in diverse ways to their family and environment, and is generally conflicting. Families do not plan or prepare for having a child who will depend on their care, to a greater or lesser degree, for their entire life, causing them to experience, for a time, the feeling of symbolic death or the death of the idealized child^(21,23,24,28,29)^.

Just as we grieve the death of a loved one or close friend, the symbolic grief over the death of an idealized child can occur at any time, from diagnosis during pregnancy, after birth, or during the child’s growth and development, since at some point signs and symptoms will appear that will reveal their clinical condition. In these cases, it is common for feelings such as frustration, anxiety, and rejection of the child by one or both parents to arise, in addition to the fear of not knowing how to care for the child and not being able to cope with all the resources necessary for their upbringing and health maintenance. Added to this is the insecurity stemming from the need to manage the multiple resources required for the creation and maintenance of their health, including the correct handling and administration of medications, as well as the appropriate use of assistive equipment and technologies^(15,21,22,23,28,29,30,31,32,33,38,39)^.

This confusion of experienced sensations tends, in many cases, to destabilize marriages, with the perception of a lack of parental mutuality, and favor the onset or worsening of minor mental disorders such as anxiety, stress, and depression, leading to spousal abandonment, divorce, and the weakening of significant family social networks. This leaves full-time care to mothers and older siblings, when families have more children^(18,23,28,37,43,44,49)^.

This family structure, now matriarchal in nature, fosters the emergence of other needs, such as finding a job, or a better occupation than the current one, and in many cases, even a second job, which may include, even if only partially, coverage from a supplementary health insurance plan. Furthermore, it becomes necessary to reorganize one’s own significant social network, which is often weakened by the daily demands of caregiving^(18,23,28,37,43,44,49,50)^.

Given the difficulty and, in some cases, inability to obtain a better job or to afford the supplementary assistance of a health plan, these new families are becoming heavily dependent on social and pension benefits to supplement their income. In many cases, this condition results in a high dependence on public health and education policies, which become essential to minimally meet their daily needs. Programs such as the “Farmácia Popular” (Popular Pharmacy), Bolsa Família (Family Allowance), cooking gas aid, the Continuous Benefit Payment (*BPC*), and high-cost medications and supplies are benefits commonly requested by families with children and/or adolescents with disabilities or neurodevelopmental disorders, in addition to other resources such as the Brazilian Social Assistance Reference Center (*CRAS*) and the Specialized Social Assistance Reference Center (*CREAS*)^(22,23,25,27,29,35,36,44)^.

Even when families manage to go through the process of accepting and adapting to the new reality imposed with all the changes, adjustments, and health demands that become part of daily life, one of the biggest challenges reported still lies in the multiple barriers imposed socially. These barriers manifest themselves in various ways: Urban planning issues – such as uneven or cracked sidewalks, which hinder the safe and autonomous movement of children and adolescents who use wheelchairs or have visual impairments; communication and informational issues – such as the absence of professionals trained in Libras (Brazilian Sign Language) or the lack of books in Braille in schools and health units; architectural issues – such as the lack of access ramps and handrails in health services and schools, bathrooms with narrow doors, making them unusable for people with disabilities; attitudinal issues – such as not receiving invitations to children’s parties and other social events, the use of derogatory terms such as “disabled” or “poor thing,” or disrespect for autonomy, such as asking the caregiver, instead of the blind child/adolescent, if they are hungry; methodological issues – such as the refusal of school enrollment due to a lack of qualified professionals or the absence of pedagogical resources adapted to their needs^(33,34,44,49,50)^.

A particularly sensitive point addressed in the selected studies is the process of exclusion and invisibility reported by families of children and/or adolescents with disabilities or neurodevelopmental disorders, which permeates all aspects of their lives. These children and adolescents are considered to have less efficient bodies, containing less essence than bodies considered normative, suffering from exclusionary and ableist practices relativized by strongly defended neoliberal policies. The materiality of their bodies can no longer be hidden, yet they remain marginalized by society, being exposed to prejudice, violence, digital influence, substance abuse, and crime^(15,16,17,20,21,22,23,24,26,29,32,35,37,38,39,50,51)^.

No less important, studies also point to the difficulty of access that these families experience when trying to access primary health care, represented in this study by structural barriers – health units without access ramps, with bathrooms that do not allow the passage of a wheelchair – and attitudinal barriers, mainly. Families report that services are geared towards the child’s/adolescent’s disability, forgetting that this child/adolescent is more than just the disability or clinical condition present, because in addition to specific needs, they have the same needs as other children considered ‘normal’^(15,17,18,19,20,21,22,33,34,35,43,44,52,53)^.

These findings align with other evidence pointing to weaknesses in the organization of Primary Health Care, especially in territories marked by greater vulnerability. A study based on PMAQ-AB data revealed significant disparities in the work processes of primary health care teams between urban, rural, and remote municipalities, showing that the absence of systematic planning, poor territorialization, and unequal population coverage compromise the effectiveness of health actions, especially in the most distant and underserved areas^(54)^.

Studies also report a lack of professionals within the primary care units who act as a reference for these families. When specialized care is required, people encounter long and lengthy waiting lines, creating significant bottlenecks in the healthcare system. This issue was particularly exacerbated during the Covid-19 pandemic, when the gap in communication and coordination between primary and secondary care levels was intensified, further hindering access to continuous and adequate care. As a result, many of these children and adolescents stopped receiving the necessary support. The healthcare professionals in the assigned units have little or no training on care for people with disabilities or neurodevelopmental disorders, resulting in a low rate of resolution of their requirements^(18,22,30,31,32,34,38,39,41,43,44,55,56,57,58,59)^.

### Aspects that Weaken the Performance of Primary Health Care

When the Brazilian Public Health System (*SUS*) was created in 1990, PHC was conceived and developed to achieve the highest possible degree of decentralization and capillarity, to ensure universal, equitable, comprehensive, continuous, longitudinal, and quality care to a population assigned within a specific territory. Its entry point is the Primary Health Units (*UBS*), and its main working tool is knowledge of the potentials and weaknesses of that territory through the creation of bonds with the population served^(19,20,23,30,31,37,38,40,41,53,55,56,57,58,59)^.

**Figure 4:**
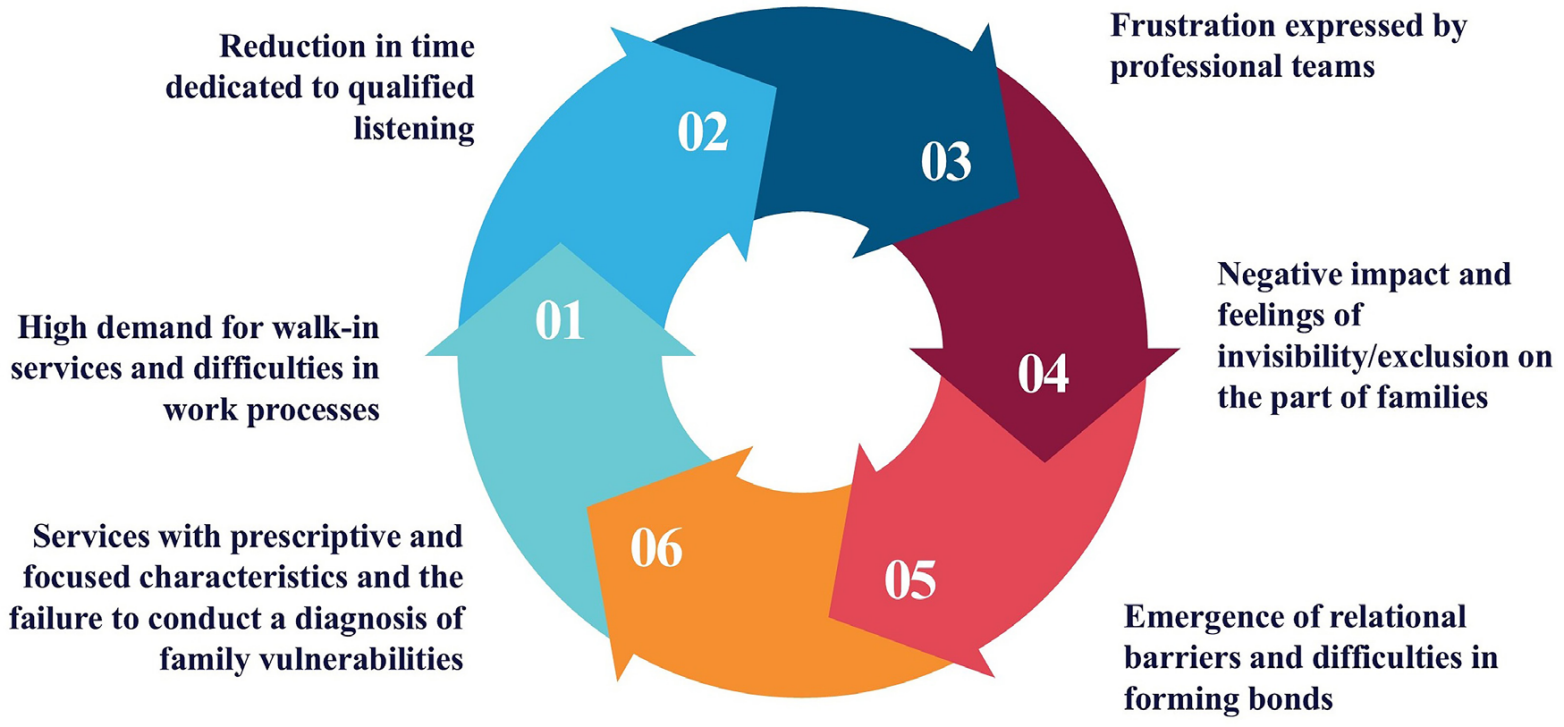
Weaknesses presented by PHC in providing care to families of children and/or adolescents with disabilities or neurodevelopmental disorders – Pelotas, RS, 2025.

However, that’s not what we find. Studies indicate that PHC has great potential to become a reality. Nevertheless, even though it had all its attributes during its conception and creation, this same PHC fails to fully exploit its potential and proves to be quite fragile in terms of the quality of care and attention given to the families of children and adolescents with disabilities or neurodevelopmental disorders, which can be easily explained by the diagram presented^(60,61,62,63)^ (Figure 4).

Despite the advances proposed by the National Policy for Comprehensive Child Health Care – *PNAISC* ^(64)^ and by the Care Network for People with Disabilities – *RCPD* ^(65)^, the findings of this review demonstrate that PHC still lacks effective and comprehensive action in monitoring the families of children and adolescents with disabilities or neurodevelopmental disorders. There is a noticeable disconnection between normative guidelines and daily practice, especially regarding the capacity of PHC teams to implement family-centered, longitudinal, and interdisciplinary care, as advocated by these policies^(64,65,66,67)^.

One of the main elements weakening PHC performance is related to the insufficient early identification of risk signs for child development. Although technical materials exist, such as the Child Development Monitoring Guides^(68)^, the Protocol for Attention to Children with Warning Signs for Neuropsychomotor Development^(69)^ and the Manuals for Caring for Children with Autism Spectrum Disorders^(67)^, this review highlights that such devices are poorly understood and underutilized by primary care teams. This gap results in missed windows of opportunity for early interventions, significantly compromising prognoses and increasing the burden on families, who are forced to seek specialized services late, often outside their own area^(25,27,43)^.

Additionally, some detachment is observed between PHC and the organizational principles of the *RCPD*, especially regarding intersectoral coordination and the development of care pathways that guarantee continuous and qualified access to services. Families report fragmented pathways, marked by disjointed therapeutic itineraries, a lack of clear referral and counter-referral flows, and difficulties in accessing Specialized Rehabilitation Centers (*CER*s), when they exist in the area. This fragility highlights the partial functioning of the network, which ends up transferring the responsibility for managing care to families themselves^(15,41,44)^.

Another recurring aspect in the studies is the inadequacy in the training of primary health care professionals to work with children and adolescents with disabilities and neurodevelopmental disorders. This inadequacy directly impacts the quality of the practices developed, which remain anchored in biomedical, prescriptive models focused on clinical conditions, to the detriment of approaches centered on promoting development, strengthening family skills, and guaranteeing rights^(19,23,35)^. This scenario reveals the urgent need for ongoing health education processes, aligned with the PNAISC guidelines^(64)^ and the contents of the Manuals for Healthcare for People with Disabilities^(68,69)^, which emphasize the centrality of the family in care and the collaborative role of health, education, and social assistance.

The limited effectiveness of development monitoring actions, coupled with the low utilization of instruments such as the Early Childhood Legal Framework^(66)^, the Guidelines for Healthcare for Children with Neurodevelopmental Disorders^(67,69)^, and the benchmarks of the PNAISC itself reveal that PHC performance remains insufficient in the face of these families’ complex needs. This not only weakens the early detection of health problems, but also widens inequalities in access, since a significant portion of interventions ends up being shifted to medium and high complexity services – contradicting the organizational principles of the network and overburdening socially vulnerable families^(24,34,55)^.

Furthermore, a significant gap is observed in PHC performance for adolescents with disabilities and/or neurodevelopmental disorders. The findings of this review indicate that primary care actions tend to focus on early childhood, neglecting the specific needs of this transitional population, which requires continuous interdisciplinary approaches sensitive to the life cycle. Added to this is the scarcity of specific clinical guidelines aimed at these populations within the scope of PHC, which weakens professional practice, restricts the comprehensiveness of care, and compromises the coordination of actions between the various levels of the health system.

Given this context, it becomes essential to rethink work processes in PHC, so that practices are reorganized in light of care centered on the family, the community, and human development. The effective implementation of the PNAISC and RCPD guidelines requires that health units systematically incorporate developmental surveillance actions, qualified home visits, the construction of individualized therapeutic projects, and coordinated action with the intersectoral network. This implies not only technical training, but also cultural changes within the services, capable of deconstructing ableist and biomedical practices, thus promoting a truly inclusive, effective, and equity-promoting PHC^(27,44,67)^.

## CONCLUSION

This review reinforces the personal, family, and social impacts that a diagnosis of disability and/or neurodevelopmental disorder has on families, causing them to suffer on various levels, whether from the feeling of anticipatory grief experienced due to the diagnosis, or from the stigmatization and exclusion still perceptible in society. It highlights the negative repercussions of these diagnoses, the lack of preparedness in managing and caring for these children/adolescents, and the [re]organization of family configurations caused, in most cases, by abandonment by spouses, making mothers the sole caregivers of the diagnosed children. This reinforces the reported difficulty in accessing PHC and the dependence these families have on social security and welfare programs and benefits.

The review also points out that primary health care professionals have limited technical skills in addressing children/adolescents with disabilities and/or neurodevelopmental disorders and their families, resulting in isolated actions focused solely on the health condition, with prescriptive and focused approaches – a biomedical model of care exclusively directed to the child, when not to their needs. The difficulty faced by healthcare teams and units in establishing consistent bonds with these families is evident, as is the imperative need to recognize them as central units of care. The proactive role of healthcare teams, especially nursing staff, becomes essential in strengthening these bonds, to promote the development of family potential and allow for the accurate identification of health needs. Moreover, professionals demonstrate a superficial knowledge of fundamental concepts such as integrated care networks, longitudinal care, care pathways, and the use of PTS (Personalized Treatment Plan) and VD (Home Visit), in addition to a lack of knowledge or unfamiliarity with specific legislation aimed at supporting this population.

The findings of this review show that, although Primary Health Care plays a strategic role in the care of families of children and adolescents with disabilities or neurodevelopmental disorders, important gaps persist related to the training of teams, qualified listening to families, and intersectoral coordination. Overcoming these limitations requires recognizing these children as subjects of rights and fostering shared responsibility among the different levels of care and sectors involved. Ensuring continuous, comprehensive, and respectful care involves understanding the diversity of family experiences and building practices that are sensitive to their needs, not limited to the clinical condition, but affirming their dignity and citizenship.

Furthermore, few studies acknowledge the leading role of children and adolescents with disabilities or neurodevelopmental disorders themselves in the care process, which contradicts the principle of active participation advocated by the disability rights movement, summarized in the slogan “Nothing about us without us”. This absence deserves to be problematized and addressed in PHC services, in research, and in the formulation of public policies.

## Data Availability

The entire dataset supporting the results of this study was published in the article itself.
