# Supplementary material for: Primary care services role in supporting families of children or adolescents with disabilities or neurodevelopmental disorders
Source: Rev Esc Enferm USP. 2026 Feb 16;60:e20250152. doi: 10.1590/1980-220X-REEUSP-2025-0152en (PMC12930535; doi:10.1590/1980-220X-REEUSP-2025-0152en)
Supplement: Translation

# Translation: Atuação da atenção primária às famílias de crianças ou adolescentes com deficiência ou transtornos do neurodesenvolvimento*

**DOI:** 10.1590/1980-220X-REEUSP-2025-0152pt

**Authors:** Luciano Silveira Pacheco de Medeiros, Thiago Ferreira Abreu, Viviane Marten Milbrath, Ruth Irmgard Bärtschi Gabatz, Sônia Silva Marcon, Luciano Garcia Lourenção, Giovana Calcagno Gomes, Clarice Alves Bonow

**Affiliations:** 1 Universidade Federal de Pelotas, Faculdade de Enfermagem, Programa de Pós-Graduação em Enfermagem, Pelotas, RS, Brasil.; 2 Universidade Estadual de Maringá, Programa de Pós-Graduação em Enfermagem, Maringá, PR, Brasil.; 3 Universidade Federal do Rio Grande, Escola de Enfermagem, Programa de Pós-Graduação em Enfermagem, Rio Grande, RS, Brasil.

**Keywords:** Cuidados de enfermagem, Crianças com deficiência, Transtornos do Neurodesenvolvimento, Família, Atenção Primária à Saúde

## Abstract

**Objetivo:**

Mapear e sintetizar as práticas, estratégias e intervenções de cuidado desenvolvidas na Atenção Primária à Saúde para as famílias de crianças e adolescentes com deficiência e/ou transtornos do neurodesenvolvimento.

**Método:**

Revisão de escopo conduzida segundo as diretrizes do Joanna Briggs Institute e a checklist PRISMA-ScR. Foram consultadas as bases LILACS, IBECS, Scielo, CINAHL, WoS, MEDLINE/PubMed, Scopus e PsycINFO, com inclusão de estudos publicados entre 2015 e 2024, nos idiomas português, inglês e espanhol.

**Resultados:**

Foram incluídos 31 estudos, que permitiram a construção de duas categorias temáticas: sentimentos experienciados e dinâmica das famílias; e aspectos que fragilizam a atuação da APS. Evidenciaram-se dificuldades de acesso, modelo de cuidado prescritivo, escuta limitada das famílias e formação técnica insuficiente dos profissionais.

**Conclusão:**

A Atenção Primária à Saúde ainda apresenta lacunas importantes para o cuidado integral e equitativo às famílias de crianças com deficiência e/ou transtornos do neurodesenvolvimento. Superá-las exige escuta qualificada, articulação intersetorial e reconhecimento dessas famílias como núcleo central do cuidado.

## INTRODUÇÃO

A sociedade moderna vive mais e com melhor qualidade de vida. Como benefícios da modernidade podemos citar todas as tecnologias que auxiliam a humanidade em suas tarefas cotidianas, proporcionando, dessa maneira, mais tempo para outras atividades que não aquelas necessárias à subsistência. Porém, todas essas facilidades disponíveis contribuem significativamente para o aumento da inatividade física, do número de pessoas com doenças crônicas não transmissíveis (DNCT), com deficiências físicas adquiridas e/ou comorbidades associadas, dentre outras^(1)^. Vive-se mais, porém muitas vezes em condições não muito saudáveis, o que resulta em pessoas dependentes de cuidados de média e longa permanência em hospitalizações frequentes e até mesmo em mortes evitáveis e precoces^(1)^.

O aumento da expectativa de vida globalmente experenciado, associado às descobertas e inovações tecnológicas na assistência à saúde, contribui para o crescimento do grupo de pessoas com essas condições. Estudos realizados nos Estados Unidos, por exemplo, apontam que 18,5% das crianças e adolescentes com idades entre zero e 17 anos apresentam alguma deficiência, transtorno do neurodesenvolvimento ou alguma necessidade especial de saúde - grupo também conhecido pela sigla CRIANES – e que necessitarão de cuidados complexos e contínuos de saúde em longo prazo. Isso implicará em diversas internações hospitalares, uso de tecnologias assistivas para proporcionar melhor qualidade de vida e, ainda, acompanhamento por profissionais da Atenção Primária à Saúde – APS^(2,3)^.

No Brasil, embora faltem dados epidemiológicos atualizados sobre essa população, o Instituto Brasileiro de Geografia e Estatística (IBGE) identificou, no Censo de 2010, aproximadamente 45,6 milhões de pessoas com deficiência, sendo cerca de 760 mil crianças e/ou adolescentes^(2,3)^. Ainda que tais dados careçam de atualizações sistemáticas, são indicativos de um cenário de grande relevância para a formulação de políticas públicas e práticas de cuidado.

A Organização Mundial da Saúde (OMS) define deficiência como um termo abrangente que inclui deficiências físicas, mentais, intelectuais ou sensoriais de longo prazo, que, em interação com diversas barreiras, podem obstruir a participação plena e efetiva na sociedade em igualdade de condições com as demais pessoas. Já os transtornos do neurodesenvolvimento referem-se a condições que se manifestam precocemente e afetam o funcionamento pessoal, social ou acadêmico das crianças, exigindo cuidados multidisciplinares e contínuos. A Lei Brasileira de Inclusão da Pessoa com Deficiência (LBI – Lei nº 13.146/2015), alinhada à Convenção da ONU sobre os Direitos das Pessoas com Deficiência (Decreto nº 6.949/2009), reafirma o direito à saúde, à participação social e à dignidade dessas populações, convocando os serviços de saúde a oferecerem cuidado equitativo, integral e acessível.

A Convenção sobre os Direitos das Pessoas com Deficiência, aprovada pela Organização das Nações Unidas em 2006, foi incorporada ao ordenamento jurídico brasileiro com equivalência constitucional, nos termos do §3º do artigo 5º da Constituição Federal. Junto à Lei Brasileira de Inclusão (Lei nº 13.146/2015), constitui o principal marco legal para garantia do direito à saúde integral e à participação social das pessoas com deficiência.

Não faz muito que, para que cada um dos papéis fosse devidamente desempenhado dentro da sociedade - sendo o homem provedor e a mulher cuidadora - não se admitia o nascimento de crianças diferentes^(4)^. Crianças com deficiência ou alguma doença crônica, assim como os idosos, já não são tão frequentemente deixadas para trás em detrimento da sobrevivência dos demais do grupo como outrora. No entanto, a experiência da maternidade/paternidade de crianças com deficiências ou transtornos do neurodesenvolvimento ainda é uma experiência que tende a impactar negativamente a dinâmica familiar, sendo frequente o surgimento de crises^(5)^. A dinâmica familiar mostra-se alterada, surgem novas demandas de saúde, situações de capacitismo e exclusão tornam-se corriqueiras, suas redes de apoio tendem a tornar-se frágeis, o que pode levar ao sentimento de luto pelo filho vivo, ou seja, aquele sentimento de perda que os genitores tendem a sentir mesmo na presença do seu filho^(5,6,7,8)^.

É importante destacar que, muitas vezes, os serviços de saúde ainda operam sob uma lógica biomédica e patologizante, centrada na doença e não na pessoa, o que reforça a exclusão de crianças com deficiência e invisibiliza o protagonismo de suas famílias. Essa perspectiva corponormativa precisa ser criticamente analisada para que se possa avançar na construção de práticas de cuidado mais equitativas, intersetoriais e centradas no sujeito.

Nesse cenário, a APS, por ser a principal porta de entrada do Sistema Único de Saúde (SUS) e por estar mais próxima do cotidiano das famílias, deve ser o primeiro espaço de acolhimento, cuidado longitudinal e construção de vínculos com essas populações. De acordo com a Organização Pan-Americana da Saúde (OPAS), cabe à APS identificar e responder, de forma contínua e oportuna, às necessidades das pessoas e comunidades, priorizando ações centradas na pessoa, com base em princípios de equidade, integralidade e justiça social.

Com base no exposto, esse estudo teve por objetivo mapear e sintetizar as práticas, estratégias e intervenções de cuidado desenvolvidas na Atenção Primária à Saúde para as famílias de crianças e adolescentes com deficiência e/ou transtornos do neurodesenvolvimento.

## MÉTODO

### Tipo de Estudo

Trata-se de uma revisão de escopo^(9)^, conduzida de acordo com as diretrizes metodológicas do *Joanna Briggs Institute - Manual for Evidence Synthesis e Preferred Reporting Items for Systematic reviews and Meta-Analyses extension for Scoping Reviews (PRISMA-ScR) Checklist*. As revisões de escopo têm como propósito mapear e sintetizar as evidências atuais disponíveis na literatura, de modo a destacar as lacunas de conhecimento em relação a determinado fenômeno, incitando a realização de novas pesquisas^(10)^. O protocolo dessa revisão está registrado na plataforma Open Science Framework (OSF) sob o DOI n. https://doi.org/10.17605/OSF.IO/Q4NSZ.

Em sua realização foram seguidos os passos metodológicos propostos, a saber: a) identificação da questão de pesquisa, b) identificação de estudos relevantes, c) seleção dos estudos, d) mapeamento dos dados, e, e) coleta, resumo e relato dos resultados^(10)^. Considerando a estratégica de busca utilizada a partir do acrônimo PCC (População, Conceito, Contexto), foi elaborada a seguinte questão norteadora de pesquisa: “Quais são as práticas, estratégias e intervenções de cuidado disponíveis na literatura nacional e internacional, desenvolvidas na APS, voltadas às famílias de crianças e adolescentes com deficiência e/ou transtornos do neurodesenvolvimento?”, onde, P (População): Famílias de crianças e adolescentes com deficiência e/ou transtornos do neurodesenvolvimento; C (Conceito): Práticas, estratégias e intervenções que caracterizam a atuação de cuidado; e, C (Contexto): APS.

### Coleta de Dados

Para a extração das informações foram consultadas, durante o meses de janeiro e fevereiro de 2025, as publicações indexadas nas bases de dados da Literatura Latino-Americana e do Caribe em Ciências da Saúde (LILACS), Índice Bibliográfico Espanhol de Ciências da Saúde (IBECS), *Scientific Electronic Library Online* (Scielo), *Current Index to Nursing and Allied Health Literature* (CINAHL), *Web of Science* (WoS), *Medical Literature Analysis and Retrieval System Online* (MEDLINE/ Pubmed), *SciVerse Scopus* (Scopus), *American Psychological Association* (PsycINFO).

Os critérios de inclusão foram estudos originais disponibilizados na íntegra, resultantes de pesquisas qualitativas, quantitativas ou estudos mistos, nos idiomas português, inglês e espanhol.

A delimitação linguística aos idiomas português, inglês e espanhol justifica-se por serem os mais amplamente utilizados nas bases de dados selecionadas e corresponderem às línguas dominadas pela equipe de pesquisadores. Além disso, essas línguas contemplam a maior parte da produção científica relevante na área da saúde nas Américas, conforme demonstrado em revisões sistemáticas anteriores.

Para a exclusão foram considerados: trabalhos publicados antes de 2015 e trabalhos duplicados. O recorte temporal a partir de 2015 justifica-se pela promulgação da Lei Brasileira de Inclusão da Pessoa com Deficiência (LBI – Lei n.º 13.146/2015)^(11)^, que instituiu diretrizes normativas, éticas e organizacionais fundamentais para a consolidação dos direitos das pessoas com deficiência no Brasil. Essa legislação influenciou diretamente as práticas e políticas de saúde no âmbito da Atenção Primária.

### Estratégia de Busca

Foi utilizada a mesma estratégia de busca em todas as bases consultadas, com o auxílio dos operadores booleanos AND e OR, utilizando-se os Descritores em Ciências da Saúde (DeCS) e *Medical Subject Headings* (MeSH): ((Family OR Caregivers) AND (“disabled children” OR adolescent OR “Neurodevelopmental Disorders” OR “Persons with Mental Disabilities”) AND “Primary Health Care” AND (“Nursing Care” OR “Nurses, Public Health” OR Nursing)).

A presente revisão optou por não incluir literatura cinzenta entre as fontes de busca, priorizando estudos disponíveis em bases indexadas com critérios de seleção mais homogêneos e padronizados. Essa escolha visou garantir maior rastreabilidade, comparabilidade metodológica e reprodutibilidade dos resultados. Reconhece-se, entretanto, que a literatura não indexada pode conter contribuições relevantes ao campo e que sua exclusão constitui uma limitação a ser considerada na discussão dos achados.

A seleção dos estudos deu-se em três etapas: leitura dos títulos, leitura dos resumos e leitura dos artigos na íntegra, e o processo foi realizado, de forma simultânea, por dois autores independentes, garantindo-se o cegamento. Não houve discordâncias em nenhuma das etapas.

Para a etapa de separação, sumarização e relatórios dos resultados encontrados foi utilizado um instrumento estruturado que permitiu a extração dos seguintes dados: título, autores, ano de publicação, delineamento, objetivo, participantes, local de realização, instituição proponente, periódico de publicação e principais resultados.

### Análise e Tratamento dos Dados

Para análise e tratamento dos dados, realizou-se inicialmente uma síntese descritiva com o levantamento das principais características dos estudos selecionados. Em seguida, procedeu-se a uma segunda síntese, de natureza categórica, na qual foram destacados os principais achados desses estudos. Nessa etapa utilizou-se o *Interface de R pour les Analyses Multidimensionnelles de Textes et de Questionnaires* (IRAMUTEQ), versão 0.8 alpha 7, por meio da técnica de proximidade léxica, ou seja, pelo agrupamento de textos semelhantes em seções, com consequente identificação de temas comuns, por meio da Classificação Hierárquica Descendente (CHD), também conhecida como método de Reinert^(12)^. As categorias que emergiram após o uso do software foram discutidas sob as lentes da Análise Crítica do Discurso (ACD)^(13)^.

A análise dos dados textuais foi realizada com auxílio do software IRAMUTEQ, por meio da Classificação. Embora o dendrograma seja um recurso visual relevante nessa técnica, a representação gráfica gerada originalmente não pôde ser recuperada, razão pela qual não foi incluída neste manuscrito. As informações extraídas por meio da CHD, no entanto, foram descritas textualmente e orientaram a categorização dos achados.

### Aspectos Éticos

Por se tratar de um estudo de revisão de escopo, não houve a necessidade de submissão e apreciação da proposta por um Comitê de Ética em Pesquisa (CEP).

## RESULTADOS

No processo de identificação, foram localizados 1.207 estudos primários nas bases de dados consultadas: 216 (17,9%) na Scopus, 118 (9,8%) na WoS, 465 (38,5%) na MEDLINE/Pubmed, 13 (1,1%) na IBECS, 90 (7,4%) na LILACS, 79 (6,5%) na CINAHAL, 159 (13,2%) na PsycINFO e, ainda, 67 (5,6%) por meio de buscas nas citações e referências dos trabalhos excluídos nesta etapa - artigos de revisão.

A Figura 1 ilustra os processos e etapas percorridas para a seleção dos estudos encontrados, conforme recomendações PRISMA^(10)^.

**Figura 1:** Processo de elegibilidade dos estudos encontrados conforme recomendações do PRISMA^(10)^ – Pelotas, RS, 2025.

Na etapa de triagem foram excluídos 1.176 estudos: 613 (52,1%) estudos pelo critério ano, 123 (10,4%) pelo critério da disponibilidade, 41 (3,5%) por serem estudos de revisão e manuais, 27 (2,3%) por serem duplicados, 369 (31,4%) por estarem foram do escopo proposto e três (0,3%) por serem preprints, restando 31 estudos elegíveis para as discussões. Quanto à abordagem utilizada nos estudos, temos 24 (77,4%) qualitativos^(14,15,16,17,18,19,20,21,22,23,24,25,26,27,28,29,30,31,32)^, seis (19,4%) quantitativos^(33,34,35,36,37,38)^ e um (3,2%) estudo de métodos mistos^(39)^; destes, 18 (58,1%) estudos foram publicados em revistas nacionais^(14,15,17,18,20,21,22,23,24,25,26,33,35,40,41,42,43,44)^ e 13 (41,9%) em revistas internacionais^(20,28,29,30,31,32,33,34,36,37,38,39,45)^, de nacionalidades variadas. Com relação aos países de origem dos estudos, 21 (67,7%) publicados no Brasil^(14,15,16,17,18,19,20,21,22,23,24,25,26,33,34,35,40,44)^, um (3,2%) no Chile^(36)^, um (3,2%) no Canadá^(28)^, três (9,8%) nos Estados Unidos^(29,30,37)^, um (3,2%) na Espanha^(38)^, um (3,2%) na Coréia do Sul^(31)^, um (3,2%) na Arábia Saudita^(39)^, e dois (6,5%) na Austrália^(32,45)^, conforme mostra a Figura 2.

**Figura 2:** Distribuição espacial, por países, dos estudos selecionados – Pelotas, RS, 2025.

Dos 31 estudos incluídos nesta revisão, sete abordaram exclusivamente condições associadas à deficiência (como paralisia cerebral, deficiência física ou intelectual), enquanto 11 focalizaram apenas os transtornos do neurodesenvolvimento (TND), a exemplo do transtorno do espectro autista e transtorno do déficit de atenção com hiperatividade (TDAH). Outros dez estudos contemplaram simultaneamente aspectos de deficiência e TND, evidenciando a sobreposição conceitual e clínica entre essas condições. Três estudos não apresentaram informações suficientes para uma categorização precisa, por tratarem de temas transversais ou enfoques mais amplos. Essa distribuição permite identificar tendências na produção científica e reforça a importância de diferenciar conceitualmente os grupos analisados, uma vez que as demandas familiares e os modos de cuidado desenvolvidos na APS variam conforme o diagnóstico predominante.

Nos estudos que abordaram exclusivamente a deficiência, observou-se predominância de práticas centradas na reabilitação funcional da criança, com foco em encaminhamentos especializados, uso de dispositivos assistivos e orientações pontuais voltadas ao cuidado físico e à dependência de tecnologias de suporte. Nesses casos, a APS é frequentemente acionada como porta de entrada ou ponto de referência para obtenção de benefícios e recursos materiais. Por outro lado, nos estudos que trataram apenas de transtornos do neurodesenvolvimento, como o transtorno do espectro autista, houve maior ênfase em ações voltadas ao apoio psicossocial das famílias, à articulação com serviços de saúde mental e educação, e à continuidade do cuidado. Nos estudos que contemplaram ambas as condições, os achados evidenciam práticas mais integradas, embora ainda marcadas por lacunas no acompanhamento longitudinal e na escuta das famílias. A análise sugere que o tipo de condição predominante influencia o foco das ações desenvolvidas na APS, o que demanda preparo técnico e sensibilidade para reconhecer a complexidade dos contextos familiares envolvidos.

A maioria dos estudos incluídos focaliza crianças em idade pré-escolar e escolar, com predominância de faixas etárias entre zero e doze anos. Observou-se escassez de estudos que abordem especificamente adolescentes com deficiência ou transtornos do neurodesenvolvimento no contexto da Atenção Primária à Saúde. Ainda que alguns artigos incluam adolescentes em suas amostras, poucos desenvolvem análises específicas sobre as particularidades dessa fase da vida. Essa lacuna etária é mencionada por alguns autores como uma limitação metodológica, sobretudo diante da necessidade de garantir continuidade do cuidado e estratégias adaptadas ao processo de transição da infância para a adolescência. A ausência de foco específico nos adolescentes aponta para um desafio assistencial relevante e para a necessidade de ampliar o olhar da APS sobre essa população.

A maior parte dos estudos analisados refere-se ao modelo da Estratégia Saúde da Família (ESF), predominante no cenário brasileiro, com descrição das atividades realizadas por enfermeiros, médicos e agentes comunitários. Também foram identificados estudos que mencionam a atuação de Unidades Básicas de Saúde convencionais e, em menor número, serviços de atenção primária organizados de forma integrada ao ambiente escolar ou vinculados a políticas locais específicas, como observado em experiências do Chile, da Espanha e da Austrália. Apesar da diversidade de arranjos, poucos estudos exploram de forma aprofundada como o modelo de organização da APS influencia na qualidade e na continuidade do cuidado oferecido às famílias de crianças com deficiência ou transtornos do neurodesenvolvimento. Essa ausência analítica representa uma lacuna na literatura e reforça a importância de considerar os contextos institucionais na avaliação das práticas em saúde.

Embora os estudos tenham sido conduzidos em diferentes regiões do Brasil e em outros países, a maioria não descreve com profundidade as especificidades regionais dos contextos analisados, o que constitui uma limitação para a compreensão das dinâmicas locais de organização e acesso aos serviços de atenção primária.

Com relação ao idioma, prevalecem os artigos publicados em inglês (21–67,7%)^(16,17,18,19,20,26,28,29,30,31,32,34,36,37,38,39,41,44)^, nove (29,1%) em português^(15,16,17,21,22,23,35,41,43,44)^ e um (3,2%) em espanhol^(36)^. Em relação ao ano de publicação, um (3,3%) artigo no ano de 2015^(29)^, três (9,7%) em 2017^(15,16,33)^, quatro (12,9%) em 2018^(17,28,31,32)^, quatro (12,9%) em 2019^(18,19,20,34)^, sete (22,6%) em 2020^(21,35,40,41,42,43,44)^, quatro (12,9%) em 2021^(22,23,37,39)^, dois (6,4%) em 2022^(24,45)^, dois (6,4%) em 2023^(25,36)^ e, quatro (12,9%) em 2024^(26,27,30,38)^ (Figura 3).

**Figura 3:** Principais características dos estudos selecionados – Pelotas, RS, 2025.

Observa-se forte rede colaborativa entre docentes pesquisadores de programas de pós-graduação de universidades públicas federais, principalmente no eixo sul-sudeste-centro-oeste^(18,19,21,22,23,24,25,26,27,33,35,41,42,43,44)^. Isso pode ser justificado pelo fato de o Brasil ter um sistema de saúde único, público e gratuito, com cobertura nacional, com a capacidade de oferecer uma resposta minimamente satisfatória às demandas de saúde apresentadas pelos usuários, e que tem como proposta a porta de entrada na Atenção Primária à Saúde (APS) e a resolução das demandas dos usuários, quando possível, dentro do seu território de origem^(46,47)^.

Aliado a esse fato, temos uma rede de instituições superiores de ensino públicas federais com programas de graduação e pós-graduação de excelência, com diversas ações de ensino, pesquisa e extensão, com vistas a formar e qualificar profissionais aptos a uma assistência integral e humanizada ao binômio sujeito-família^(48)^.

A fim de se possibilitar a discussão dos resultados observados nos estudos selecionados, os pontos foram agrupados num quadro sinóptico: sentimentos experienciados e dinâmica das famílias; e aspectos que fragilizam a atuação da APS (Quadro 1).

**Quadro 1:** Determinantes sociais de saúde e aspectos que influenciam o cuidado na Atenção Primária à Saúde (APS) – Pelotas, RS, Brasil, 2025.

Sentimentos experienciados e dinâmica das famílias	
Dimensões observadas	Descrição dos achados
Sentimentos negativos com a descoberta do diagnóstico	‘E agora?’. ‘O que eu vou fazer?’. ‘Por que comigo?’ ‘Como eu vou cuidar?’. ‘Meu marido vai aceitar?’. ‘Como eu vou pagar os tratamentos?’, são apenas algumas das perguntas que vêm à cabeça das famílias quando do momento do diagnóstico de uma deficiência ou da possibilidade de um transtorno do neurodesenvolvimento de um filho; as famílias sentem-se despreparadas para enfrentar o cenário que agora se apresenta diante delas^(18,19,27,34,36,41,43)^. Angústia, medo, tristeza, sentimento de luto pelo filho vivo são alguns dos sentimentos que se percebe, o que impacta negativamente em todas as dimensões da dinâmica familiar com consequente diminuição da sensação de bem-estar e instalação e agravamento de transtornos mentais menores^(15,21,22,23,28,31,32,33,37,38,39)^.
Processo de invisibilização e exclusão das crianças e adolescentes com transtornos do neurodesenvolvimento ou deficiência e suas famílias	As pessoas com alguma deficiência ou transtorno do neurodesenvolvimento já passaram por atravessamentos de diversas naturezas ao longo da história. Atualmente sofrem com as constantes castrações em seus processos de subjetivação e consequente invisibilização e alienação, delas e de suas famílias^(23,25,34,41)^. Crianças e adolescentes com transtornos do neurodesenvolvimento ou deficiência são considerados como corpos menos eficientes e contendo menos essência que os corpos considerados normativos, sofrendo com práticas excludentes e capacitistas relativizadas pelas políticas neoliberais fortemente defendidas. A materialidade de seus corpos não pode mais ser escondida, porém, segue colocada à margem da sociedade, expondo-os ao preconceito, exposição a violências, influência digital, uso de substâncias psicoativas e criminalidade^(15,16,18,22,28,29,30,31,32,33,36,38,39,43,44)^.
Predominância da mulher como principal cuidadora da família	Predominância da mulher enquanto cuidadora principal na família e percepção de não mutualidade parental, acarretando em diminuição da sensação de bem-estar e qualidade de vida referidas, fragilidades em suas redes sociais significativas e até mesmo existência delas, impactando diretamente na instalação e/ou agravamento de quadros de transtornos mentais menores, tais como ansiedade, stress e depressão, o que tende a levar a separações e divórcios, acentuando ainda mais a dinâmica familiar já alterada^(17,19,22,25,28,32,37,40,43,45)^.
Desconhecimento ou despreparo da família em como utilizar medicamentos, equipamentos e dispositivos de tecnologias assistivas	A manipulação e administração regular de medicamentos, o uso de equipamentos e tecnologias assistivas acompanham, de forma geral, o diagnóstico de uma deficiência ou transtorno do neurodesenvolvimento^(21,22,24,25,35,41,43,44)^. Essas famílias, na maioria das vezes gerenciada pelas mães, constantemente referem desconhecimento e níveis bastante elevados de medo e despreparo para a realização de atividades, agora necessárias, para a manutenção do estado de saúde de seus filhos, fato esse que aumenta a dependência do suporte e auxilio em longo prazo de profissionais minimamente capacitados, representados, no imaginário dessas famílias, na figura dos profissionais de enfermagem^(15,16,17,19,21,22,24,25,30,31,33,35,38,40,43,44)^.
Dependência de benefícios sociais e previdenciários	Os contextos socioeconômicos e culturais apresentados pelas famílias são, em grande parte, complexos e resultado de baixa escolaridade dos pais associada às condições de renda, emprego, moradia, e acesso a serviços de saúde, o que impacta negativamente nas condições de vida dessas famílias, fazendo com que necessitem cada vez mais dos benefícios assistenciais sociais e previdenciários e dependência quase que absoluta das políticas públicas de educação e saúde^(17,18,19,20,33,34,40,44)^.
Dificuldades de acessibilidade	Os estudos sinalizam as diversas barreiras de acessibilidade na Atenção Primária à Saúde percebidas pelas famílias, dentre as quais podem-se citar as barreiras estruturais, específicas das edificações das unidades de saúde responsáveis pelas famílias adscritas nos territórios, as barreiras atitudinais, representadas pela estigmatização, preconceito e capacitismo percebidos pelas famílias durante e para que os atendimentos aconteçam^(15,16,18,19,21,23,24,25,33,34,36,40,41,44,45)^. Essas famílias também relatam as constantes peregrinações pelas redes de Atenção Primária à Saúde, subutilização, baixa resolutividade e a necessidade da complementação dos seus serviços com a contratação de planos de saúde privados^(15,16,18,19,21,22,23,30,34,39,40,41,44)^.
**Aspectos que fragilizam a atuação da APS**	
**Dimensões observadas**	**Descrição dos achados**
Criação e fortalecimento de vínculo com as famílias	A criação e o fortalecimento de vínculos dos profissionais de enfermagem com as famílias assistidas é considerada um importante e potente ferramenta^(19,20,27,29,36,40,41)^, pois possibilita o [re]conhecimento da realidade em que essas famílias estão inseridas, seus territórios de adscrição e dos seus determinantes sociais de saúde de formas mais ampla e contextualizada; porém, observam-se vínculos frágeis entre os profissionais de enfermagem e as famílias sob sua responsabilidade, fato esse responsável por atendimentos pontuais e conhecimento raso da realidade vivida pelas famílias^(17,18,21,22,24,26,30,32,33,35,37,42,43,44)^.
Subutilização das visitas domiciliares e projeto terapêutico singular e baixa efetividade das ações educativas em saúde	Observou-se a subutilização de ações educativas para a promoção de práticas em saúde, principalmente aquelas desenvolvidas durante as visitas domiciliares. As ações educativas dispensadas pela enfermagem mostraram-se restritas ao ensino do uso de materiais, equipamentos e medicamentos^(15,17,18,19,20,33,35,40,43)^, não considerando a família enquanto um sistema, aberto e fechado, em constantes interações com o meio ambiente, o que lhe possibilita a troca de informações e de recursos necessários para as suas demandas^(49,50,51)^, necessidade que se faz da assistência de enfermagem com o deslocamento do cuidado centrado no modelo biomédico para o modelo social da deficiência com olhar, também, nos transtornos do neurodesenvolvimento com a família como ‘foco’ e longitudinalidade da assistência^(22,23,24,26,28,29,30,31,32,37,39)^.
Trabalho articulado com os níveis especializados de média e alta complexidade e demais redes colaborativas	As demandas de saúde apresentadas concentram-se, em grande parte, nos serviços e tecnologias disponibilizados na Unidade Básica de Saúde (UBS) referenciada para seu território de adscrição; porém, em diversas situações, faz-se necessário o encaminhamento dessas famílias para os níveis especializados de média e alta complexidade^(17,21,25,26,27,28,36,41)^ e a necessidade do diálogo constante e horizontalizado com Assistência Social, Educação e demais redes colaborativas identificadas como importantes para as famílias, dentro do seu território de adscrição, com a determinação de linhas de cuidados, fluxos de atendimentos, itinerários terapêuticos, elaboração do Projeto Terapêutico Singular (PTS) e o fortalecimento do sistema de referência e contrarreferência (SCR)^(15,16,18,23,34,39,45)^.

A primeira categoria aborda todas as determinantes sociais de saúde e suas implicações diretas nas demandas de saúde das famílias com crianças e adolescentes com transtornos do neurodesenvolvimento ou deficiência: sentimentos negativos com a descoberta do diagnóstico; processo de invisibilização e exclusão das crianças e adolescentes com transtornos do neurodesenvolvimento ou deficiência e suas famílias; predominância da mulher como principal cuidadora da família; desconhecimento ou despreparo da família em como utilizar medicamentos, equipamentos e dispositivos de tecnologias assistivas; dependência de benefícios sociais e previdenciários; dificuldades de acessibilidade.

Na segunda categoria são discutidos os aspectos relacionados com quantidade e qualidade da assistência na APS junto às famílias de crianças e adolescentes com transtornos do neurodesenvolvimento ou deficiência: criação e fortalecimento de vínculo com as famílias; subutilização das visitas domiciliares e baixa efetividade das ações educativas em saúde; trabalho articulado com os níveis especializados de média e alta complexidade e demais em redes colaborativas.

## DISCUSSÃO

A discussão a seguir retoma os elementos organizados no Quadro 1, que sintetiza os principais determinantes sociais de saúde e aspectos que influenciam o cuidado na APS com famílias de crianças e adolescentes com deficiência e/ou TND. A análise foi estruturada em dois eixos interpretativos derivados da síntese dos achados: a) Sentimentos experenciados e dinâmica das famílias; e, b) Aspectos que fragilizam a atuação da APS.

### Sentimentos Experienciados e Dinâmica das Famílias

O diagnóstico de uma deficiência ou transtorno do neurodesenvolvimento de uma criança e/ou adolescente pode se apresentar de maneiras diversas para sua família e seu entorno e são, geral, conflituosas. As famílias não planejam e nem se preparam para ter um filho que dependerá de seus cuidados, em maior ou em menor grau, para a vida inteira, fazendo com que experimentem, por algum tempo, o sentimento de morte simbólica ou morte do filho idealizado^(21,23,24,28,29)^.

Assim como ocorre no luto que sentimos quando da morte concreta de algum ente querido ou amigo próximo, o sentimento de luto simbólico pela morte do filho idealizado pode acontecer a qualquer momento, desde o diagnóstico ainda durante a gestação, a partir do nascimento ou durante o crescimento e desenvolvimento desse filho, já que, em algum momento, aparecerão sinais e sintomas que evidenciarão a sua condição clínica. Nestes casos, é comum o surgimento de sentimentos como frustração, ansiedade, rejeição da criança, por um ou ambos os genitores, além do medo de não saber cuidar do filho e de não conseguir lidar com todos os recursos necessários para sua criação e manutenção de seu estado de saúde. Soma-se a isso a insegurança diante da necessidade de lidar com múltiplos recursos necessários para sua criação e manutenção da saúde, incluindo a manipulação e administração correta de medicamentos, bem como o uso apropriado de equipamentos e tecnologias assistivas^(15,21,22,23,28,29,30,31,32,33,38,39)^.

Essa confusão de sensações experenciadas, tendem, em muitos casos, a desestabilizar os casamentos, com a percepção de não mutualidade parental, e favorecer a instalação ou o agravamento de quadros de transtornos mentais menores, tais como ansiedade, stress e depressão, ocasionando o abandono dos cônjuges, o divórcio e também o enfraquecimento das redes sociais significativas familiares, deixando, dessa maneira, o cuidado, em tempo integral, para as mães e para o irmãos mais velhos, quando as famílias já tem mais filhos^(18,23,28,37,43,44,49)^.

Essa apresentação familiar, agora de conformação matriarcal, favorece o aparecimento de outras demandas, tais como encontrar um emprego, ou uma ocupação melhor do que a atual e, em muitos casos, até um segundo emprego, que incluam, ainda que parcialmente, a cobertura de um plano de saúde suplementar. Além disso, torna-se necessário [re]organizar sua própria rede social significativa, muitas vezes fragilizada diante das exigências cotidianas do cuidado^(18,23,28,37,43,44,49,50)^.

Diante da dificuldade e, em alguns casos, incapacidade de obter um emprego melhor ou de arcar com a complementação assistencial de um plano de saúde, observa-se que essas novas famílias passam a depender fortemente de benefícios sociais e previdenciários para complementarem suas rendas. Em muitos casos, essa condição resulta em uma elevada dependência das políticas públicas de saúde e educação, que se tornam essenciais para atender, minimamente, as suas demandas cotidianas. Programas como farmácia popular, bolsa família, auxílio gás, benefício de prestação continuada – BPC, medicamentos e insumos de alto custo são benefícios comumente solicitados por famílias com crianças e/ou adolescentes com deficiência ou transtorno do neurodesenvolvimento, além de outros dispositivos como o Centro de Referência de Assistência Social (CRAS) e o Centro de Referência Especializado de Assistência Social (CREAS)^(22,23,25,27,29,35,36,44)^.

Mesmo quando as famílias conseguem passar pelo processo de aceitação e adequação da nova realidade imposta com todas as mudanças, ajustes e demandas de saúde que passam a fazer parte do cotidiano, um dos maiores desafios relatados ainda reside nas múltiplas barreiras impostas socialmente. Essas barreiras manifestam-se de diversas formas: urbanísticas – como calçadas irregulares ou com falhas, o que dificulta o deslocamento seguro e autônomo de crianças e adolescentes com mobilidade em cadeira de rodas ou com deficiência visual; comunicacionais e informacionais – como a ausência de profissionais habilitados em Libras ou a falta de livros em braile nas escolas e unidade de saúde; arquitetônicas – como a inexistência de rampas de acesso e corrimão em serviços de saúde e escolas, banheiros com portas estreitas, inviabilizando o uso por pessoas com deficiência; atitudinais – como o não recebimento de convites para festas infantis e outros eventos sociais, o uso de termos pejorativos como “deficiente” ou “coitadinho”, ou, ainda, o desrespeito à autonomia, como quando se pergunta ao acompanhante, em vez da criança/adolescente cegos, por exemplo, se eles estão com fome; metodológicas – como a recusa de matrícula escolar por falta de profissionais capacitados ou inexistência de recursos pedagógicos adaptados às necessidades desses^(33,34,44,49,50)^.

Ponto bastante sensível abordado nos estudos selecionados é o processo de exclusão e de invisibilização relatado pelas famílias de crianças e/ou adolescentes com deficiência ou transtornos do neurodesenvolvimento que transversaliza todos os aspectos de suas vidas. Essas crianças e adolescentes são considerados como corpos menos eficientes e contendo menos essência que os corpos considerados normativos, sofrendo com práticas excludentes e capacitistas relativizadas pelas políticas neoliberais fortemente defendidas. A materialidade de seus corpos não pode mais ser escondida, porém, segue colocada à margem da sociedade, expondo-os ao preconceito, à violências, influência digital, uso de substâncias psicoativas e criminalidade^(15,16,17,20,21,22,23,24,26,29,32,35,37,38,39,50,51)^.

Não menos importante, os estudos apontam, também, a dificuldade de acesso que essas famílias experimentam quando tentam acessar a APS, representadas, nesse estudo, por barreiras estruturais – unidades de saúde sem rampa de acesso, com banheiros que não permitem a passagem de uma cadeira de rodas, e atitudinais, principalmente. As famílias referem que os atendimentos são direcionados para a deficiência da criança/adolescente, esquecendo-se que essa criança/adolescente é mais do que a deficiência ou condição clínica ali presente, pois para além das necessidades específicas apresentam as mesmas necessidades que as demais crianças, consideradas ‘normais’^(15,17,18,19,20,21,22,33,34,35,43,44,52,53)^.

Esses achados alinham-se a outras evidências, que apontam fragilidades na organização da Atenção Primária à Saúde, especialmente em territórios marcados por maior vulnerabilidade. Um estudo baseado em dados do PMAQ-AB evidenciou disparidades significativas no processo de trabalho das equipes da APS entre municípios urbanos, rurais e remotos, revelando que a ausência de planejamento sistemático, a territorialização precária e a cobertura populacional desigual comprometem a efetividade das ações de saúde, sobretudo nas áreas mais distantes e desassistidas^(54)^.

Os estudos também relatam a falta de profissionais dentro das unidades básicas que atuam como referência dessas famílias. Quando há necessidade de atendimento especializado deparam-se com filas de espera extensas e demoradas, configurando importantes gargalos no sistema de saúde. Esse aspecto foi particularmente agravado durante a pandemia de Covid-19, quando se intensificou o hiato na comunicação e articulação entre os níveis de atenção primária e secundária, o que dificultou ainda mais o acesso a cuidados contínuos e adequados. Como consequência, muitas dessas crianças e adolescentes deixaram de receber o acompanhamento necessário. Os profissionais de saúde das unidades adscritas possuem pouco ou nenhum preparo para o atendimento de pessoas com deficiência ou transtornos do neurodesenvolvimento, com consequente baixa da resolutividade das demandas que apresentam^(18,22,30,31,32,34,38,39,41,43,44,55,56,57,58,59)^.

### Aspectos que Fragilizam a Atuação da APS

Quando da criação do SUS, no ano de 1990, a APS foi pensada e desenvolvida para que atingisse o maior grau de descentralidade e capilaridade possíveis, a fim de garantir, a uma população adscrita dentro de um território específico, atendimento universal, equânime, integral, contínuo, longitudinal e de qualidade, tendo como sua porta de entrada as Unidades Básicas de Saúde (UBS) e como principal ferramenta de trabalho o conhecimento das potencialidades e das fragilidades desse território por meio da criação de vínculos com a população atendida^(19,20,23,30,31,37,38,40,41,53,55,56,57,58,59)^.

Mas não é isso que encontramos. Os estudos apontam uma APS com grande potencial de vir a ser. Porém, mesmo que imbuída de todos seus atributos quando da sua ideação e criação, essa mesma APS não consegue explorar toda a sua potencialidade e mostra-se bastante fragilizada no que tange à qualidade dos atendimentos e o cuidado às famílias de crianças e adolescentes com deficiência ou distúrbio do neurodesenvolvimento, o que pode facilmente ser explicado pelo diagrama apresentado^(60,61,62,63)^ (Figura 4).

**Figura 4:** Fragilidades apresentadas pela APS no atendimento às famílias de crianças e/ou adolescentes com deficiência ou transtornos do neurodesenvolvimento – Pelotas, RS, 2025.

Apesar dos avanços propostos pela Política Nacional de Atenção Integral à Saúde da Criança - PNAISC^(64)^ e pela Rede de Cuidados à Pessoa com Deficiência – RCPD^(65)^, os achados desta revisão demonstram que a APS ainda carece de uma atuação efetiva e integral no acompanhamento das famílias de crianças e adolescentes com deficiência ou transtornos do neurodesenvolvimento. Nota-se uma desconexão entre as diretrizes normativas e a prática cotidiana, sobretudo no que se refere à capacidade das equipes de APS em operacionalizar o cuidado centrado na família, longitudinal e interdisciplinar, conforme preconizado por essas políticas^(64,65,66,67)^.

Um dos principais elementos que fragilizam a atuação da APS está relacionado à insuficiência na identificação precoce dos sinais de risco para o desenvolvimento infantil. Embora existam materiais técnicos, como os Guias de Vigilância do Desenvolvimento Infantil^(68)^, o Protocolo de Atenção à Criança com Sinais de Alerta para o Desenvolvimento Neuropsicomotor^(69)^ e os Manuais de Atenção às Crianças com Transtornos do Espectro Autista^(67)^, a presente revisão evidencia que tais dispositivos são pouco conhecidos e subutilizados pelas equipes da atenção básica. Essa lacuna resulta na perda de janelas de oportunidade para intervenções precoces, comprometendo significativamente os prognósticos e aumentando a sobrecarga das famílias, que se veem obrigadas a buscar tardiamente serviços especializados, muitas vezes fora do seu território^(25,27,43)^.

Adicionalmente, observa-se um distanciamento entre a APS e os princípios organizativos da RCPD, especialmente no que se refere à articulação intersetorial e à construção de linhas de cuidado que garantam o acesso contínuo e qualificado aos serviços. As famílias relatam percursos fragmentados, marcados por itinerários terapêuticos desarticulados, ausência de fluxos claros de referência e contrarreferência, além de dificuldades no acesso aos Centros Especializados em Reabilitação (CER), quando existentes no território. Essa fragilidade evidencia um funcionamento parcial da rede, que acaba por transferir para as famílias a responsabilidade pela gestão dos próprios cuidados^(15,41,44)^.

Outro aspecto recorrente nos estudos é a insuficiência na formação dos profissionais de APS para atuar com crianças e adolescentes com deficiência e transtornos do neurodesenvolvimento. Tal insuficiência impacta diretamente na qualidade das práticas desenvolvidas, que permanecem ancoradas em modelos biomédicos, prescritivos e focalizados na condição clínica, em detrimento de abordagens centradas na promoção do desenvolvimento, no fortalecimento das competências familiares e na garantia de direitos^(19,23,35)^. Esse cenário revela a necessidade urgente de processos permanentes de educação em saúde, alinhados às diretrizes da PNAISC^(64)^ e aos conteúdos dos Manuais de Atenção à Saúde da Pessoa com Deficiência^(68,69)^, os quais enfatizam a centralidade da família no cuidado e a atuação colaborativa entre saúde, educação e assistência social.

A pouca efetividade das ações de vigilância do desenvolvimento, somada à baixa utilização de instrumentos como o Marco Legal da Primeira Infância^(66)^, os Guias para Atenção à Saúde de Crianças com Transtornos do Neurodesenvolvimento^(67,69)^ e os referenciais da própria PNAISC, denuncia uma atuação da APS que segue insuficiente frente às necessidades complexas dessas famílias. Isso não apenas fragiliza a detecção precoce de agravos, mas também amplia as desigualdades no acesso, uma vez que parte significativa das intervenções acaba sendo deslocada para os serviços de média e alta complexidade - contrariando os princípios organizativos da rede e sobrecarregando famílias socialmente vulnerabilizadas^(24,34,55)^.

Além disso, observa-se uma lacuna significativa na atuação da APS junto ao público adolescente com deficiência e/ou transtornos do neurodesenvolvimento. Os achados desta revisão apontam que as ações da atenção primária tendem a se concentrar na primeira infância, deixando de lado as especificidades dessa população em fase de transição, que demanda abordagens interdisciplinares contínuas e sensíveis ao ciclo de vida. Soma-se a isso a escassez de diretrizes clínicas específicas voltadas a esses públicos no âmbito da APS, o que fragiliza a atuação profissional, restringe a integralidade do cuidado e compromete a coordenação das ações entre os diversos níveis do sistema de saúde.

Diante desse contexto, torna-se imprescindível repensar os processos de trabalho na APS, de modo que as práticas sejam reorganizadas à luz do cuidado centrado na família, na comunidade e no desenvolvimento humano. A implementação efetiva das diretrizes da PNAISC e da RCPD exige que as unidades de saúde incorporem, de forma sistemática, as ações de vigilância do desenvolvimento, visitas domiciliares qualificadas, construção de projetos terapêuticos singulares e atuação articulada com a rede intersetorial. Isso implica não apenas na formação técnica, mas também em mudanças culturais no interior dos serviços, capazes de desconstruir práticas capacitistas e biomédicas, promovendo, assim, uma APS verdadeiramente inclusiva, resolutiva e promotora de equidade^(27,44,67)^.

## CONCLUSÃO

Essa revisão reforça os impactos pessoal, familiar e social que um diagnóstico de deficiência e/ou transtorno do neurodesenvolvimento tem sobre as famílias, fazendo com que sofram em diversos níveis, seja pelo sentimento de luto antecipado experenciado pelo diagnóstico, seja pelo movimento de estigmatização e exclusão ainda perceptíveis na sociedade. Evidencia as repercussões negativas desses diagnósticos, o despreparo no manejo e cuidado dessas crianças/adolescentes e a [re]organização das configurações familiares ocasionadas, em sua maioria, pelo abandono dos cônjuges, fazendo com que as mães sejam as únicas responsáveis pela criação dos filhos diagnosticados. Reforça a dificuldade relatada de acesso à APS e a dependência que essas famílias apresentam dos programas e benefícios previdenciários e sociais.

A revisão, aponta, ainda, profissionais de saúde atuantes na APS com pouca habilidade técnica para a abordagem de crianças / adolescentes com deficiência e/ou transtornos do neurodesenvolvimento e suas famílias, o que resulta em ações pontuais pautadas somente na condição de saúde, com ações prescritivas e focativas - modelo biomédico de assistência voltado exclusivamente para a criança, quando não para suas necessidades. Evidencia-se a dificuldade das equipes e unidades de saúde em estabelecer vínculos consistentes com essas famílias, além da necessidade imperativa de reconhecê-las como unidades centrais do cuidado. Torna-se essencial a atuação proativa das equipes de saúde, principalmente da enfermagem, no fortalecimento desses vínculos, de modo a favorecer o desenvolvimento das potencialidades familiares e permitir a identificação precisa das necessidades de saúde. Observa-se, ainda, um conhecimento superficial por parte dos profissionais sobre conceitos fundamentais como atenção integrada em redes, longitudinalidade dos cuidados, linhas de cuidado e uso do PTS e VD, além do desconhecimento ou pouca familiaridade com a legislação específica voltada ao suporte dessa população.

Os achados desta revisão evidenciam que, embora a Atenção Primária à Saúde desempenhe um papel estratégico no cuidado às famílias de crianças e adolescentes com deficiência ou transtornos do neurodesenvolvimento, persistem lacunas importantes relacionadas à formação das equipes, à escuta qualificada das famílias e à articulação intersetorial. Superar essas limitações requer o reconhecimento dessas crianças como sujeitos de direitos e a corresponsabilização dos diferentes níveis de atenção e setores envolvidos. Garantir o cuidado contínuo, integral e respeitoso passa por compreender a diversidade de experiências familiares e construir práticas sensíveis às suas necessidades, que não se limitem à condição clínica, mas afirmem sua dignidade e cidadania.

Além disso, poucos estudos reconhecem o protagonismo das próprias crianças e adolescentes com deficiência ou transtornos do neurodesenvolvimento no processo de cuidado, o que contraria o princípio da participação ativa defendido pelo movimento das pessoas com deficiência, sintetizado na bandeira “Nada sobre nós sem nós”. Essa ausência merece ser problematizada e enfrentada nos serviços de APS, na pesquisa e na formulação de políticas públicas.

## Data Availability

Todo o conjunto de dados que dá suporte aos resultados deste estudo foi publicado no próprio artigo.

# Translation: El papel de los servicios de atención primaria en el apoyo a las familias de niños o adolescentes con discapacidades o trastornos del neurodesarrollo*

**DOI:** 10.1590/1980-220X-REEUSP-2025-0152es

**Authors:** Luciano Silveira Pacheco de Medeiros, Thiago Ferreira Abreu, Viviane Marten Milbrath, Ruth Irmgard Bärtschi Gabatz, Sônia Silva Marcon, Luciano Garcia Lourenção, Giovana Calcagno Gomes, Clarice Alves Bonow

**Affiliations:** 1 Universidade Federal de Pelotas, Faculdade de Enfermagem, Programa de Pós-Graduação em Enfermagem, Pelotas, RS, Brasil.; 2 Universidade Estadual de Maringá, Programa de Pós-Graduação em Enfermagem, Maringá, PR, Brasil.; 3 Universidade Federal do Rio Grande, Escola de Enfermagem, Programa de Pós-Graduação em Enfermagem, Rio Grande, RS, Brasil.

**Keywords:** Atención de Enfermería, Niños con Discapacidad, Trastornos del Neurodesarrollo, Familia, Atención Primaria de Salud

## Abstract

**Objetivo:**

Mapear y sintetizar las prácticas, estrategias e intervenciones de cuidado desarrolladas en Atención Primaria de Salud para familias de niños, niñas y adolescentes con discapacidad y/o trastornos del neurodesarrollo.

**Método:**

Revisión del alcance realizada de acuerdo con las pautas del Instituto Joanna Briggs y la lista de verificación PRISMA-ScR. Se consultaron las siguientes bases de datos: LILACS, IBECS, Scielo, CINAHL, WoS, MEDLINE/PubMed, Scopus y PsycINFO, incluyendo estudios publicados entre 2015 y 2024, en portugués, inglés y español.

**Resultados:**

Se incluyeron 31 estudios, lo que permitió la construcción de dos categorías temáticas: sentimientos vividos y dinámica familiar; y aspectos que debilitan el desempeño de la atención primaria de salud. Se evidenciaron dificultades en el acceso, un modelo de atención prescriptivo, una escucha limitada a las familias y una formación técnica insuficiente de los profesionales.

**Conclusión:**

La atención primaria de salud aún presenta importantes brechas a la hora de brindar atención integral y equitativa a las familias de niños con discapacidades y/o trastornos del neurodesarrollo. Superar estos desafíos requiere una escucha experta, coordinación intersectorial y el reconocimiento de estas familias como foco central de la atención.

## INTRODUCCIÓN

La sociedad moderna vive más y con mejor calidad de vida. Entre los beneficios de la modernidad podemos citar todas las tecnologías que ayudan a la humanidad en sus tareas cotidianas, proporcionando así más tiempo para otras actividades que no sean las necesarias para la subsistencia. Sin embargo, todas estas facilidades disponibles contribuyen significativamente al aumento de la inactividad física, del número de personas con enfermedades crónicas no transmisibles (ECNT), con discapacidades físicas adquiridas y/o comorbilidades asociadas, entre otras^(1)^. Se vive más, pero a menudo en condiciones poco saludables, lo que da lugar a personas dependientes de cuidados de media y larga duración, hospitalizaciones frecuentes e incluso muertes evitables y prematuras^(1)^.

El aumento de la esperanza de vida experimentado a nivel mundial, asociado a los descubrimientos e innovaciones tecnológicas en la asistencia sanitaria, contribuye al crecimiento del grupo de personas con estas condiciones. Estudios realizados en Estados Unidos, por ejemplo, indican que el 18,5% de los niños y adolescentes de entre 0 y 17 años presentan alguna discapacidad, trastorno del desarrollo neurológico o alguna necesidad especial de salud —grupo también conocido por la sigla CRIANES— y que necesitarán cuidados de salud complejos y continuos a largo plazo. Esto implicará varias hospitalizaciones, el uso de tecnologías de asistencia para proporcionar una mejor calidad de vida y, además, el seguimiento por parte de profesionales de la Atención Primaria de Salud (APS)^(2,3)^.

En Brasil, aunque faltan datos epidemiológicos actualizados sobre esta población, el Instituto Brasileiro de Geografia e Estatística (IBGE) identificó, en el Censo de 2010, aproximadamente 45,6 millones de personas con discapacidad, de las cuales alrededor de 760 000 eran niños y/o adolescentes^(2,3)^. Aunque estos datos carecen de actualizaciones sistemáticas, son indicativos de un escenario de gran relevancia para la formulación de políticas públicas y prácticas de atención.

La Organización Mundial de la Salud (OMS) define la discapacidad como un término amplio que incluye discapacidades físicas, mentales, intelectuales o sensoriales de larga duración que, en interacción con diversas barreras, pueden obstaculizar la participación plena y efectiva en la sociedad en igualdad de condiciones con las demás personas. Por su parte, los trastornos del desarrollo neurológico se refieren a afecciones que se manifiestan de forma precoz y afectan al funcionamiento personal, social o académico de los niños, lo que requiere cuidados multidisciplinarios y continuos. La Ley Brasileña de Inclusión de las Personas con Discapacidad (LBI - Ley n.° 13.146/2015), en consonancia con la Convención de las Naciones Unidas sobre los Derechos de las Personas con Discapacidad (Decreto n.° 6.949/2009), reafirma el derecho a la salud, la participación social y la dignidad de estas poblaciones, instando a los servicios de salud a ofrecer una atención equitativa, integral y accesible.

La Convención sobre los Derechos de las Personas con Discapacidad, aprobada por las Naciones Unidas en 2006, se incorporó al ordenamiento jurídico brasileño con equivalencia constitucional, en los términos del párrafo 3 del artículo 5 de la Constitución Federal. Junto con la Ley Brasileña de Inclusión (Ley n.° 13.146/2015), constituye el principal marco legal para garantizar el derecho a la salud integral y a la participación social de las personas con discapacidad.

No hace mucho tiempo, para que cada uno de los roles se desempeñara adecuadamente dentro de la sociedad —el hombre como proveedor y la mujer como cuidadora—, no se admitía el nacimiento de niños diferentes^(4)^. Los niños con discapacidad o alguna enfermedad crónica, al igual que los ancianos, ya no son abandonados con tanta frecuencia en detrimento de la supervivencia del resto del grupo como antes. Sin embargo, la experiencia de la maternidad/paternidad de niños con discapacidades o trastornos del desarrollo neurológico sigue siendo una experiencia que tiende a afectar negativamente a la dinámica familiar, siendo frecuente la aparición de crisis^(5)^. La dinámica familiar se ve alterada, surgen nuevas demandas de salud, las situaciones de capacitismo y exclusión se vuelven habituales, sus redes de apoyo tienden a fragilizarse, lo que puede llevar al sentimiento de duelo por el hijo vivo, es decir, ese sentimiento de pérdida que los padres tienden a sentir incluso en presencia de su hijo^(5,6,7,8)^.

Es importante destacar que, a menudo, los servicios de salud siguen funcionando bajo una lógica biomédica y patologizante, centrada en la enfermedad y no en la persona, lo que refuerza la exclusión de los niños con discapacidad y invisibiliza el protagonismo de sus familias. Esta perspectiva corponormativa debe ser analizada críticamente para poder avanzar en la construcción de prácticas de cuidado más equitativas, intersectoriales y centradas en el sujeto.

En este contexto, la APS, al ser la principal puerta de entrada al Sistema Único de Salud (SUS) y estar más cerca de la vida cotidiana de las familias, debe ser el primer espacio de acogida, atención longitudinal y construcción de vínculos con estas poblaciones. Según la Organización Panamericana de la Salud (OPS), corresponde a la APS identificar y responder, de manera continua y oportuna, a las necesidades de las personas y las comunidades, priorizando las acciones centradas en la persona, basadas en los principios de equidad, integralidad y justicia social.

Sobre la base de lo anterior, el objetivo de este estudio fue mapear y sintetizar las prácticas, estrategias e intervenciones de atención desarrolladas en la Atención Primaria de Salud para las familias de niños y adolescentes con discapacidad y/o trastornos del desarrollo neurológico.

## MÉTODO

### Tipo de Estudio

Se trata de una revisión de alcance^(9)^, realizada de acuerdo con las directrices metodológicas del *Joanna Briggs Institute - Manual for Evidence Synthesis y Preferred Reporting Items for Systematic reviews and Meta-Analyses extension for Scoping Reviews (PRISMA-ScR) Checklist*. Las revisiones de alcance tienen como objetivo mapear y sintetizar la evidencia actual disponible en la literatura, con el fin de destacar las lagunas de conocimiento en relación con un fenómeno determinado, lo que incita a realizar nuevas investigaciones^(10)^. El protocolo de esta revisión está registrado en la plataforma Open Science Framework (OSF) con el DOI n.https://doi.org/10.17605/OSF.IO/Q4NSZ.

Para su realización se siguieron los pasos metodológicos propuestos, a saber: a) identificación de la cuestión de investigación, b) identificación de estudios relevantes, c) selección de estudios, d) mapeo de datos, y e) recolección, resumen y presentación de resultados^(10)^. Teniendo en cuenta la estrategia de búsqueda utilizada a partir del acrónimo PCC (Población, Concepto, Contexto), se elaboró la siguiente pregunta orientadora de la investigación: “¿Cuáles son las prácticas, estrategias e intervenciones de cuidado disponibles en la literatura nacional e internacional, desarrolladas en la APS, dirigidas a las familias de niños y adolescentes con discapacidad y/o trastornos del desarrollo neurológico?”, donde P (Población): Familias de niños y adolescentes con discapacidad y/o trastornos del desarrollo neurológico; C (Concepto): Prácticas, estrategias e intervenciones que caracterizan la actuación de la atención; y C (Contexto): APS.

### Recolección de Datos

Para la extracción de la información, durante los meses de enero y febrero de 2025 se consultaron las publicaciones indexadas en las bases de datos de la Literatura Latinoamericana y del Caribe en Ciencias de la Salud (LILACS), el Índice Bibliográfico Español de Ciencias de la Salud (IBECS), la *Scientific Electronic Library Online* (Scielo), *Current Index to Nursing and Allied Health Literature* (CINAHL), *Web of Science* (WoS), *Medical Literature Analysis and Retrieval System Online* (MEDLINE/ Pubmed), *SciVerse Scopus* (Scopus) y *American Psychological Association* (PsycINFO).

Los criterios de inclusión fueron estudios originales disponibles en su totalidad, resultantes de investigaciones cualitativas, cuantitativas o estudios mixtos, en los idiomas portugués, inglés y español.

La delimitación lingüística a los idiomas portugués, inglés y español se justifica por ser los más utilizados en las bases de datos seleccionadas y corresponder a los idiomas dominados por el equipo de investigadores. Además, estos idiomas abarcan la mayor parte de la producción científica relevante en el ámbito de la salud en las Américas, como se ha demostrado en revisiones sistemáticas anteriores.

Para la exclusión se consideraron: trabajos publicados antes de 2015 y trabajos duplicados. El corte temporal a partir de 2015 se justifica por la promulgación de la Ley Brasileña de Inclusión de Personas con Discapacidad (LBI – Ley n.° 13.146/2015)^(11)^, que estableció directrices normativas, éticas y organizativas fundamentales para la consolidación de los derechos de las personas con discapacidad en Brasil. Esta legislación influyó directamente en las prácticas y políticas de salud en el ámbito de la atención primaria.

### Estratégia de Busca

Se utilizó la misma estrategia de búsqueda en todas las bases consultadas, con la ayuda de los operadores booleanos AND y OR, utilizando los Descriptores en Ciencias de Salud (DeCS) e *Medical Subject Headings* (MeSH): ((Family OR Caregivers) AND (“disabled children” OR adolescent OR “Neurodevelopmental Disorders” OR “Persons with Mental Disabilities”) AND “Primary Health Care” AND (“Nursing Care” OR “Nurses, Public Health” OR Nursing)).

La presente revisión optó por no incluir literatura gris entre las fuentes de búsqueda, dando prioridad a los estudios disponibles en bases indexadas con criterios de selección más homogéneos y estandarizados. Esta elección tuvo como objetivo garantizar una mayor trazabilidad, comparabilidad metodológica y reproducibilidad de los resultados. No obstante, se reconoce que la literatura no indexada puede contener contribuciones relevantes para el campo y que su exclusión constituye una limitación que debe tenerse en cuenta en la discusión de los resultados.

La selección de los estudios se realizó en tres etapas: lectura de los títulos, lectura de los resúmenes y lectura de los artículos completos, y el proceso fue llevado a cabo simultáneamente por dos autores independientes, lo que garantizó el enmascaramiento. No hubo discrepancias en ninguna de las etapas.

Para la etapa de separación, resumen y presentación de los resultados encontrados, se utilizó un instrumento estructurado que permitió extraer los siguientes datos: título, autores, año de publicación, diseño, objetivo, participantes, lugar de realización, institución proponente, revista de publicación y principales resultados.

### Análisis y Tratamiento de los Datos

Para el análisis y tratamiento de los datos, se realizó inicialmente una síntesis descriptiva con el levantamiento de las principales características de los estudios seleccionados. A continuación, se procedió a una segunda síntesis, de naturaleza categórica, en la que se destacaron los principales hallazgos de estos estudios. En esta etapa se utilizó la *Interface de R pour les Analyses Multidimensionnelles de Textes et de Questionnaires* (IRAMUTEQ), versión 0.8 alpha 7, mediante la técnica de proximidad léxica, es decir, mediante la agrupación de textos similares en secciones, con la consiguiente identificación de temas comunes, mediante la Clasificación Jerárquica Descendente (CJD), también conocida como método de Reinert^(12)^. Las categorías que surgieron tras el uso del software se discutieron desde la perspectiva del Análisis Crítico del Discurso (ACD)^(13)^.

El análisis de los datos textuales se realizó con la ayuda del software IRAMUTEQ, mediante la Clasificación. Aunque el dendrograma es un recurso visual relevante en esta técnica, la representación gráfica generada originalmente no pudo recuperarse, por lo que no se incluyó en este manuscrito. Sin embargo, la información extraída mediante la CJD se describió textualmente y orientó la categorización de los hallazgos.

### Aspectos Éticos

Al tratarse de un estudio de revisión de alcance, no fue necesario presentar y someter la propuesta a la consideración de un Comité de Ética en Investigación (CEI).

## RESULTADOS

En el proceso de identificación, se localizaron 1207 estudios primarios en las bases de datos consultadas: 216 (17,9%) en Scopus, 118 (9,8%) en WoS, 465 (38,5%) en MEDLINE/Pubmed, 13 (1,1%) en IBECS, 90 (7,4%) en LILACS, 79 (6,5%) en CINAHAL, 159 (13,2%) en PsycINFO y, además, 67 (5,6%) mediante búsquedas en las citas y referencias de los trabajos excluidos en esta etapa: artículos de revisión.

La Figura 1 ilustra los procesos y etapas seguidos para la selección de los estudios encontrados, de acuerdo con las recomendaciones PRISMA^(10)^.

**Figura 1:** Proceso de elegibilidad de los estudios encontrados según las recomendaciones de PRISMA^(10)^ – Pelotas, RS, 2025.

En la etapa de selección se excluyeron 1176 estudios: 613 (52,1%) por el criterio del año, 123 (10,4%) por el criterio de disponibilidad, 41 (3,5%) por ser estudios de revisión y manuales, 27 (2,3%) por ser duplicados, 369 (31,4%) por estar fuera del alcance propuesto y tres (0,3%) por ser preprints, quedando 31 estudios elegibles para las discusiones. En cuanto al enfoque utilizado en los estudios, tenemos 24 (77,4%) cualitativos^(14,15,16,17,18,19,20,21,22,23,24,25,26,27,28,29,30,31,32)^, seis (19,4%) cuantitativos^(33,34,35,36,37,38)^ y un (3,2%) estudio de métodos mixtos^(39)^; de estos, 18 (58,1%) estudios se publicaron en revistas nacionales^(14,15,17,18,20,21,22,23,24,25,26,33,35,40,41,42,43,44)^ y 13 (41,9%) en revistas internacionales^(20,28,29,30,31,32,33,34,36,37,38,39,45)^, de diversas nacionalidades. En cuanto a los países de origen de los estudios, 21 (67,7%) se publicaron en Brasil^(14,15,16,17,18,19,20,21,22,23,24,25,26,33,34,35,40,44)^, uno (3,2%) en Chile^(36)^, uno (3,2%) en Canadá^(28)^, tres (9,8%) en Estados Unidos^(29,30,37)^, uno (3,2%) en España^(38)^, uno (3,2%) en Corea del Sur^(31)^, uno (3,2%) en Arabia Saudita^(39)^ y dos (6,5%) en Australia^(32,45)^, como se muestra en la Figura 2.

**Figura 2:** Distribución espacial, por países, de los estudios seleccionados – Pelotas, RS, 2025.

De los 31 estudios incluidos en esta revisión, siete abordaron exclusivamente afecciones asociadas a la discapacidad (como parálisis cerebral, discapacidad física o intelectual), mientras que 11 se centraron únicamente en los trastornos del neuro desarrollo (TND), como el trastorno del espectro autista y el trastorno por déficit de atención con hiperactividad (TDAH). Otros diez estudios contemplaron simultáneamente aspectos de la discapacidad y los TND, poniendo de manifiesto la superposición conceptual y clínica entre estas condiciones. Tres estudios no presentaron información suficiente para una categorización precisa, ya que trataban temas transversales o enfoques más amplios. Esta distribución permite identificar tendencias en la producción científica y refuerza la importancia de diferenciar conceptualmente los grupos analizados, ya que las demandas familiares y los modos de atención desarrollados en la APS varían según el diagnóstico predominante.

En los estudios que abordaron exclusivamente la discapacidad, se observó un predominio de prácticas centradas en la rehabilitación funcional del niño, con énfasis en derivaciones especializadas, uso de dispositivos de asistencia y orientaciones puntuales dirigidas al cuidado físico y la dependencia de tecnologías de apoyo. En estos casos, la APS se utiliza a menudo como puerta de entrada o punto de referencia para obtener beneficios y recursos materiales. Por otro lado, en los estudios que abordaron únicamente los trastornos del desarrollo neurológico, como el trastorno del espectro autista, se hizo mayor hincapié en las acciones orientadas al apoyo psicosocial de las familias, la articulación con los servicios de salud mental y educación, y la continuidad de la atención. En los estudios que contemplaron ambas condiciones, los hallazgos evidencian prácticas más integradas, aunque todavía marcadas por lagunas en el seguimiento longitudinal y en la escucha de las familias. El análisis sugiere que el tipo de condición predominante influye en el enfoque de las acciones desarrolladas en la APS, lo que exige preparación técnica y sensibilidad para reconocer la complejidad de los contextos familiares involucrados.

La mayoría de los estudios incluidos se centran en niños en edad preescolar y escolar, con predominio de edades comprendidas entre cero y doce años. Se observó una escasez de estudios que aborden específicamente a los adolescentes con discapacidad o trastornos del desarrollo neurológico en el contexto de la atención primaria de salud. Aunque algunos artículos incluyen a adolescentes en sus muestras, pocos desarrollan análisis específicos sobre las particularidades de esta etapa de la vida. Algunos autores mencionan esta laguna de edad como una limitación metodológica, sobre todo ante la necesidad de garantizar la continuidad de la atención y estrategias adaptadas al proceso de transición de la infancia a la adolescencia.

La ausencia de un enfoque específico en los adolescentes apunta a un reto asistencial relevante y a la necesidad de ampliar la mirada de la APS sobre esta población. La mayoría de los estudios analizados se refieren al modelo de la Estrategia de Salud Familiar (ESF), predominante en el escenario brasileño, con una descripción de las actividades realizadas por enfermeros, médicos y agentes comunitarios.

También se identificaron estudios que mencionan la actuación de las Unidades Básicas de Salud convencionales y, en menor número, los servicios de atención primaria organizados de forma integrada en el entorno escolar o vinculados a políticas locales específicas, como se observa en las experiencias de Chile, España y Australia. A pesar de la diversidad de disposiciones, pocos estudios exploran en profundidad cómo el modelo de organización de la APS influye en la calidad y la continuidad de la atención ofrecida a las familias de niños con discapacidades o trastornos del desarrollo neurológico. Esta ausencia analítica representa una laguna en la literatura y refuerza la importancia de considerar los contextos institucionales en la evaluación de las prácticas de salud.

Aunque los estudios se han realizado en diferentes regiones de Brasil y en otros países, la mayoría no describe en profundidad las especificidades regionales de los contextos analizados, lo que constituye una limitación para la comprensión de las dinámicas locales de organización y acceso a los servicios de atención primaria.

En cuanto al idioma, prevalecen los artículos publicados en inglés (21–67,7%)^(16,17,18,19,20,26,28,29,30,31,32,34,36,37,38,39,41,44)^, nueve (29,1%) en portugués^(15,16,17,21,22,23,35,41,43,44)^ y uno (3,2%) en español^(36)^. En cuanto al año de publicación, un artículo (3.3%) en 2015 (29), tres (9.7%) en 2017^(15,16,33)^, cuatro (12.9%) en 2018^(17,28,31,32)^, cuatro (12,9%) en 2019^(18,19,20,34)^, siete (22,6%) en 2020^(21,35,40,41,42,43,44)^, cuatro (12,9%) en 2021^(22,23,37,39)^, dos (6,4%) en 2022^(24,45)^, dos (6,4%) en 2023^(25,36)^ y cuatro (12,9%) en 2024^(26,27,30,38)^ (Figura 3).

**Figura 3:** Principales características de los estudios seleccionados – Pelotas, RS, 2025.

Se observa una fuerte red de colaboración entre profesores investigadores de programas de posgrado de universidades públicas federales, principalmente en el eje sur-sureste-centro-oeste^(18,19,21,22,23,24,25,26,27,33,35,41,42,43,44)^. Esto puede justificarse por el hecho de que Brasil cuenta con un sistema de salud único, público y gratuito, con cobertura nacional, con capacidad para ofrecer una respuesta mínimamente satisfactoria a las demandas de salud presentadas por los usuarios, y que tiene como propuesta la puerta de entrada a la Atención Primaria de Salud (APS) y la resolución de las demandas de los usuarios, cuando sea posible, dentro de su territorio de origen^(46,47)^.

A esto se suma una red de instituciones de educación superior públicas federales con programas de licenciatura y posgrado de excelencia, con diversas acciones de enseñanza, investigación y extensión, con el objetivo de formar y calificar a profesionales aptos para una atención integral y humanizada al binomio sujeto-familia^(48)^.

Con el fin de permitir la discusión de los resultados observados en los estudios seleccionados, los puntos se agruparon en un cuadro sinóptico: sentimientos experimentados y dinámica de las familias; y aspectos que debilitan la actuación de la APS (Cuadro 1).

**Cuadro 1:** Determinantes sociales de la salud y aspectos que influyen en la atención primaria de salud (APS) – Pelotas, RS, Brasil, 2025.

Sentimientos experimentados y dinámica de las familias	
Dimensiones observadas	Descripción de los hallazgos
Sentimientos negativos al descubrir el diagnóstico	“¿Y ahora qué?”. “¿Qué voy a hacer?”. “¿Por qué a mí?”. “¿Cómo voy a cuidar de él?”. “¿Mi marido lo aceptará?”. “¿Cómo voy a pagar los tratamientos?”, son solo algunas de las preguntas que se plantean las familias en el momento del diagnóstico de una discapacidad o de la posibilidad de un trastorno del desarrollo neurológico de un hijo; las familias se sienten desprevenidas para afrontar la situación que ahora se les presenta^(18,19,27,34,36,41,43)^. Angustia, miedo, tristeza, sentimiento de duelo por el hijo vivo son algunos de los sentimientos más e es que se perciben, lo que repercute negativamente en todas las dimensiones de la dinámica familiar, con la consiguiente disminución de la sensación de bienestar y la aparición y agravamiento de trastornos mentales me nores^(15,21,22,23,28,31,32,33,37,38,39)^.
Proceso de invisibilización y exclusión de los niños y adolescentes con trastornos del neurodesarrollo o discapacidad y sus familias	Las personas con alguna discapacidad o trastorno del desarrollo neurológico han sufrido diversos tipos de discriminación a lo largo de la historia. En la actualidad, sufren constantes castraciones en sus procesos de subjetivación y la consiguiente invisibilización y alienación, tanto de ellas mismas como de sus familias^(23,25,34,41)^. Los niños y adolescentes con trastornos del desarrollo neurológico o discapacidades son considerados cuerpos menos eficientes y con menos esencia que los cuerpos considerados normativos, y sufren prácticas excluyentes y capacitistas relativizadas por las políticas neoliberales fuertemente defendidas. La materialidad de sus cuerpos ya no puede ocultarse, pero sigue marginada de la sociedad, exponiéndolos al prejuicio, la violencia, la influencia digital, el uso de sustancias psicoactivas y la delincuencia^(15,16,18,22,28,29,30,31,32,33,36,38,39,43,44)^.
Predominio de la mujer como principal cuidadora de la familia	El predominio de la mujer como principal cuidadora de la familia y la percepción de falta de reciprocidad parental provocan una disminución de la sensación de bienestar y calidad de vida mencionadas, fragilidad en sus redes sociales significativas e incluso en su existencia, lo que repercute directamente en la aparición y/o agravamiento de trastornos mentales leves, como ansiedad, estrés y depresión, lo que tiende a provocar separaciones y divorcios, acentuando aún más la dinámica familiar ya alterada^(17,19,22,25,28,32,37,40,43,45)^.
Desconocimiento o falta de preparación de la familia sobre cómo utilizar medicamentos, equipos y dispositivos de tecnologías de asistencia	La manipulación y administración regular de medicamentos, el uso de equipos y tecnologías de asistencia acompañan, en general, al diagnóstico de una discapacidad o trastorno del desarrollo neurológico^((21,22,24,25,35,41,43,44)^. Estas familias, en su mayoría gestionadas por las madres, se refieren constantemente al desconocimiento y a los niveles bastante elevados de miedo y falta de preparación para realizar las actividades, ahora necesarias, para mantener el estado de salud de sus hijos, lo que aumenta la dependencia del apoyo y la ayuda a largo plazo de profesionales mínimamente capacitados, representados, en el imaginario de estas familias, en la figura de los profesionales de enfermería^(15,16,17,19,21,22,24,25,30,31,33,35,38,40,43,44)^.
Dependencia de las prestaciones sociales y de la seguridad social	Los contextos socioeconómicos y culturales que presentan las familias son, en gran parte, complejos y resultado del bajo nivel educativo de los padres, asociado a las condiciones de ingresos, empleo, vivienda y acceso a los servicios de salud, lo que repercute negativamente en las condiciones de vida de estas familias, haciendo que necesiten cada vez más las prestaciones asistenciales sociales y de seguridad social y que dependan casi por completo de las políticas públicas de educación y salud^(17,18,19,20,33,34,40,44)^.
Dificultades de accesibilidad	Los estudios señalan las diversas barreras de accesibilidad en la atención primaria de salud percibidas por las familias, entre las que se pueden citar las barreras estructurales, específicas de los edificios de las unidades de salud responsables de las familias inscritas en los territorios, las barreras actitudinales, representadas por la estigmatización, el prejuicio y el capacitismo percibidos por las familias durante y para que se produzcan las atenciones^(15,16,18,19,21,23,24,25,33,34,36,40,41,44,45)^. Estas familias también informan de las constantes peregrinaciones por las redes de atención primaria de salud, la infrautilización, la baja resolución y la necesidad de complementar sus servicios con la contratación de planes de salud privados^(15,16,18,19,21,22,23,30,34,39,40,41,44)^.
**Aspectos que debilitan la actuación de la APS**	
**Dimensiones observadas**	**Descripción de los hallazgos**
Creación y fortalecimiento del vínculo con las familias	La creación y el fortalecimiento de vínculos entre los profesionales de enfermería y las familias atendidas se considera una herramienta importante y potente^(19,20,27,29,36,40,41)^, ya que permite [re]conocer la realidad en la que se insertan estas familias, sus territorios de adscripción y sus determinantes sociales de salud de forma más amplia y contextualizada; sin embargo, se observan vínculos frágiles entre los profesionales de enfermería y las familias bajo su responsabilidad, lo que da lugar a una atención puntual y un conocimiento superficial de la realidad que viven las familias^(17,18,21,22,24,26,30,32,33,35,37,42,43,44)^.
Infrautilización de las visitas domiciliarias y del proyecto terapéutico singular y baja eficacia de las acciones educativas en salud.	Se observó una infrautilización de las acciones educativas para la promoción de prácticas saludables, principalmente las desarrolladas durante las visitas domiciliarias. Las acciones educativas impartidas por el personal de enfermería se limitaron a enseñar el uso de materiales, equipos y medicamentos^(15,17,18,19,20,33,35,40,43)^, sin considerar a la familia como un sistema, abierto y cerrado, en constante interacción con el medio ambiente, lo que le permite el intercambio de información y recursos necesarios para sus demandas^(49,50,51)^, necesidad que se hace de la asistencia de enfermería con el desplazamiento de la atención centrada en el modelo biomédico hacia el modelo social de la discapacidad, con una mirada también en los trastornos del neurodesarrollo con la familia como “foco” y longitudinalidad de la asistencia^(22,23,24,26,28,29,30,31,32,37,39)^.
Trabajo articulado con los niveles especializados de media y alta complejidad y otras redes colaborativas	Las demandas de salud presentadas se concentran, en gran parte, en los servicios y tecnologías disponibles en la Unidad Básica de Salud (UBS) de referencia para su territorio de adscripción; sin embargo, en diversas situaciones, es necesario derivar a estas familias a los niveles especializados de media y alta complejidad^(17,21,25,26,27,28,36,41)^ y existe la necesidad de un diálogo constante y horizontalizado con Asistencia Social, Educación y demás redes colaborativas identificadas como importantes para las familias, dentro de su territorio de adscripción, con la determinación de líneas de atención, flujos de atención, itinerarios terapéuticos, elaboración del Proyecto Terapéutico Singular (PTS) y el fortalecimiento del sistema de referencia y contrarreferencia (SCR)^(15,16,18,23,34,39,45)^.

La primera categoría aborda todos los determinantes sociales de la salud y sus implicaciones directas en las demandas de salud de las familias con niños y adolescentes con trastornos del desarrollo neurológico o discapacidad: sentimientos negativos ante el descubrimiento del diagnóstico; proceso de invisibilización y exclusión de los niños y adolescentes con trastornos del desarrollo neurológico o discapacidad y sus familias; predominio de la mujer como principal cuidadora de la familia; desconocimiento o falta de preparación de la familia sobre cómo utilizar medicamentos, equipos y dispositivos de tecnología de asistencia; dependencia de prestaciones sociales y de la seguridad social; dificultades de accesibilidad.

En la segunda categoría se discuten los aspectos relacionados con la cantidad y la calidad de la atención en la APS a las familias de niños y adolescentes con trastornos del desarrollo neurológico o discapacidad: creación y fortalecimiento del vínculo con las familias; infrautilización de las visitas domiciliarias y baja eficacia de las acciones educativas en materia de salud; trabajo articulado con los niveles especializados de media y alta complejidad y otros en redes colaborativas.

## DISCUSIÓN

La discusión que sigue retoma los elementos organizados en el Cuadro 1, que sintetiza los principales determinantes sociales de la salud y los aspectos que influyen en la atención en la APS con familias de niños y adolescentes con discapacidad y/o TND. El análisis se estructuró en dos ejes interpretativos derivados de la síntesis de los hallazgos: a) Sentimientos experimentados y dinámica de las familias; y b) Aspectos que debilitan la actuación de la APS.

### Sentimientos Experimentados y Dinámica de las Familias

El diagnóstico de una discapacidad o un trastorno del desarrollo neurológico de un niño o adolescente puede presentarse de diversas maneras para su familia y su entorno y, por lo general, es conflictivo. Las familias no planean ni se preparan para tener un hijo que dependerá de sus cuidados, en mayor o menor medida, durante toda su vida, lo que les hace experimentar, durante algún tiempo, el sentimiento de muerte simbólica o muerte del hijo idealizado^(21,23,24,28,29)^.

Al igual que ocurre con el duelo que sentimos cuando fallece un ser querido o un amigo cercano, el sentimiento de duelo simbólico por la muerte del hijo idealizado puede producirse en cualquier momento, desde el diagnóstico durante el embarazo, desde el nacimiento o durante el crecimiento y desarrollo de ese hijo, ya que, en algún momento, aparecerán signos y síntomas que evidenciarán su condición clínica. En estos casos, es común que surjan sentimientos como la frustración, la ansiedad, el rechazo del niño por parte de uno o ambos padres, además del miedo a no saber cómo cuidar al hijo y a no poder manejar todos los recursos necesarios para su crianza y el mantenimiento de su estado de salud. A esto se suma la inseguridad ante la necesidad de manejar los múltiples recursos necesarios para su crianza y mantenimiento de la salud, incluyendo la manipulación y administración correcta de medicamentos, así como el uso adecuado de equipos y tecnologías de asistencia^(15,21,22,23,28,29,30,31,32,33,38,39)^.

Esta confusión de sensaciones experimentadas tiende, en muchos casos, a desestabilizar los matrimonios, con la percepción de falta de reciprocidad parental, y favorece la aparición o el agravamiento de trastornos mentales leves, como ansiedad, estrés y depresión, lo que provoca el abandono de los cónyuges, el divorcio y también el debilitamiento de las redes sociales familiares significativas, dejando de esta manera, el cuidado a tiempo completo a las madres y a los hermanos mayores, cuando las familias ya tienen más hijos^(18,23,28,37,43,44,49)^.

Esta configuración familiar, ahora de tipo matriarcal, favorece la aparición de otras demandas, como encontrar un empleo o una ocupación mejor que la actual y, en muchos casos, incluso un segundo empleo, que incluya, aunque sea parcialmente, la cobertura de un plan de salud complementario. Además, se hace necesario [re]organizar su propia red social significativa, a menudo debilitada ante las exigencias cotidianas del cuidado^(18,23,28,37,43,44,49,50)^.

Ante la dificultad y, en algunos casos, la incapacidad de conseguir un mejor empleo o de sufragar la asistencia complementaria de un plan de salud, se observa que estas nuevas familias pasan a depender en gran medida de las prestaciones sociales y de la seguridad social para complementar sus ingresos. En muchos casos, esta situación da lugar a una elevada dependencia de las políticas públicas de salud y educación, que se vuelven esenciales para satisfacer, al mínimo, sus necesidades cotidianas. Programas como la farmacia popular, la beca familiar, la ayuda para el gas, el beneficio de prestación continua (BPC, auxilio a personas ancianas sin jubilación), los medicamentos y los insumos de alto costo son prestaciones que suelen solicitar las familias con niños y/o adolescentes con discapacidad o trastornos del desarrollo neurológico, además de otros dispositivos como el Centro de Referencia de Asistencia Social (CRAS) y el Centro de Referencia Especializado de Asistencia Social (CREAS)^(22,23,25,27,29,35,36,44)^.

Incluso cuando las familias logran pasar por el proceso de aceptación y adaptación a la nueva realidad impuesta con todos los cambios, ajustes y demandas de salud que pasan a formar parte de la vida cotidiana, uno de los mayores retos que se plantean sigue siendo las múltiples barreras impuestas socialmente. Estas barreras se manifiestan de diversas formas: urbanísticas, como aceras irregulares o con fallas, lo que dificulta el desplazamiento seguro y autónomo de niños y adolescentes en silla de ruedas o con discapacidad visual; comunicativas e informativas, como la ausencia de profesionales capacitados en lengua de señas o la falta de libros en braille en las escuelas y unidades de salud; arquitectónicas, como la inexistencia de rampas de acceso y barandillas en los servicios de salud y las escuelas, baños con puertas estrechas, lo que imposibilita su uso por parte de personas con discapacidad; actitudinales, como no recibir invitaciones a fiestas infantiles y otros eventos sociales, el uso de términos peyorativos como “discapacitado” o “pobrecito”, o incluso la falta de respeto a la autonomía, como cuando se pregunta al acompañante, en lugar del niño/adolescente ciego, por ejemplo, si tiene hambre; metodológicas: como la denegación de la matrícula escolar por falta de profesionales capacitados o la inexistencia de recursos pedagógicos adaptados a las necesidades de estos^(33,34,44,49,50)^.

Un punto muy delicado abordado en los estudios seleccionados es el proceso de exclusión e invisibilización relatado por las familias de niños y/o adolescentes con discapacidad o trastornos del desarrollo neurológico que atraviesa todos los aspectos de sus vidas. Estos niños y adolescentes son considerados como cuerpos menos eficientes y con menos esencia que los cuerpos considerados normativos, sufriendo prácticas excluyentes y capacitistas relativizadas por las políticas neoliberales fuertemente defendidas. La materialidad de sus cuerpos ya no puede ocultarse, pero sigue marginada de la sociedad, exponiéndolos al prejuicio, la violencia, la influencia digital, el uso de sustancias psicoactivas y la delincuencia^(15,16,17,20,21,22,23,24,26,29,32,35,37,38,39,50,51)^.

No menos importante, los estudios también señalan la dificultad de acceso que experimentan estas familias cuando intentan acceder a la APS, representada en este estudio por barreras estructurales —unidades de salud sin rampa de acceso, con baños que no permiten el paso de una silla de ruedas— y, principalmente, actitudinales. Las familias mencionan que la atención se centra en la discapacidad del niño/adolescente, olvidando que este niño/adolescente es más que la discapacidad o la condición clínica que presenta, ya que, además de sus necesidades específicas, tiene las mismas necesidades que los demás niños, considerados “normales”^(15,17,18,19,20,21,22,33,34,35,43,44,52,53)^.

Estos hallazgos coinciden con otras evidencias que apuntan a deficiencias en la organización de la atención primaria de salud, especialmente en territorios marcados por una mayor vulnerabilidad. Un estudio basado en datos del Programa de Mejora del Acceso y la Calidad en la Atención Primaria (PMAQ-AB) puso de manifiesto disparidades significativas en el proceso de trabajo de los equipos de APS entre municipios urbanos, rurales y remotos, revelando que la ausencia de una planificación sistemática, la precaria territorialización y la cobertura poblacional desigual comprometen la eficacia de las acciones de salud, sobre todo en las zonas más alejadas y desatendidas^(54)^.

Los estudios también informan de la falta de profesionales en las unidades básicas que actúan como referencia para estas familias. Cuando se necesita atención especializada, se enfrentan a largas y tediosas colas de espera, lo que supone importantes cuellos de botella en el sistema de salud. Este aspecto se agravó particularmente durante la pandemia de COVID-19, cuando se intensificó la brecha en la comunicación y la articulación entre los niveles de atención primaria y secundaria, lo que dificultó aún más el acceso a una atención continua y adecuada. Como consecuencia, muchos de estos niños y adolescentes dejaron de recibir el seguimiento necesario. Los profesionales de salud de las unidades adscritas tienen poca o ninguna preparación para atender a personas con discapacidad o trastornos del desarrollo neurológico, lo que se traduce en una baja resolución de las demandas que presentan^(18,22,30,31,32,34,38,39,41,43,44,55,56,57,58,59)^.

### Aspectos que Debilitan la Actuación de la APS

Cuando se creó el SUS, en 1990, la APS se concibió y desarrolló para alcanzar el mayor grado posible de descentralización y capilaridad, con el fin de garantizar a la población afiliada dentro de un territorio específico una atención universal, equitativa, integral, continua, longitudinal y de calidad, teniendo como puerta de entrada las Unidades Básicas de Salud (UBS) y como principal herramienta de trabajo el conocimiento de las potencialidades y fragilidades de ese territorio mediante la creación de vínculos con la población atendida^(19,20,23,30,31,37,38,40,41,53,55,56,57,58,59)^.

Pero no es eso lo que encontramos. Los estudios apuntan a una APS con un gran potencial por desarrollar. Sin embargo, aunque imbuida de todos sus atributos en el momento de su concepción y creación, esta misma APS no logra explotar todo su potencial y se muestra bastante frágil en lo que respecta a la calidad de la atención y el cuidado de las familias de niños y adolescentes con discapacidad o trastornos del desarrollo neurológico, lo que puede explicarse fácilmente mediante el diagrama presentado^(60,61,62,63)^ (Figura 4).

**Figura 4:** Debilidades presentadas por la APS en la atención a familias con niños y/o adolescentes con discapacidad o trastornos del desarrollo neurológico – Pelotas, RS, 2025.

A pesar de los avances propuestos por la Política Nacional de Atención Integral a la Salud de la Niñez (PNAISC por su sigla en portugués)^(64)^ y por la Red de Cuidados a las Personas con Discapacidad (RCPD)^(65)^, los resultados de esta revisión demuestran que la APS aún carece de una actuación eficaz e integral en el seguimiento de las familias de niños y adolescentes con discapacidad o trastornos del desarrollo neurológico. Se observa una desconexión entre las directrices normativas y la práctica cotidiana, sobre todo en lo que se refiere a la capacidad de los equipos de APS para poner en práctica la atención centrada en la familia, longitudinal e interdisciplinaria, tal y como recomiendan estas políticas^(64,65,66,67)^.

Uno de los principales elementos que debilitan la actuación de la APS está relacionado con la insuficiencia en la identificación temprana de los signos de riesgo para el desarrollo infantil. Aunque existen materiales técnicos, como las Guías de Vigilancia del Desarrollo Infantil^(68)^, el Protocolo de Atención al Niño con Señales de Alerta para el Desarrollo Neuropsicomotor^(69)^ y los Manuales de Atención a Niños con Trastornos del Espectro Autista^(67)^, la presente revisión evidencia que dichos dispositivos son poco conocidos y subutilizados por los equipos de atención primaria. Esta laguna da lugar a la pérdida de oportunidades para intervenciones tempranas, lo que compromete significativamente los pronósticos y aumenta la sobrecarga de las familias, que se ven obligadas a buscar tardíamente servicios especializados, a menudo fuera de su territorio^(25,27,43)^.

Además, se observa una distancia entre la APS y los principios organizativos de la RCPD, especialmente en lo que se refiere a la articulación intersectorial y la construcción de líneas de atención que garanticen un acceso continuo y cualificado a los servicios. Las familias relatan trayectorias fragmentadas, marcadas por itinerarios terapéuticos desarticulados, ausencia de flujos claros de referencia y contrarreferencia, además de dificultades para acceder a los Centros Especializados en Rehabilitación (CER), cuando existen en el territorio. Esta fragilidad pone de manifiesto un funcionamiento parcial de la red, que acaba transfiriendo a las familias la responsabilidad de la gestión de los propios cuidados^(15,41,44)^.

Otro aspecto recurrente en los estudios es la insuficiencia en la formación de los profesionales de la APS para actuar con niños y adolescentes con discapacidad y trastornos del desarrollo neurológico. Esta insuficiencia repercute directamente en la calidad de las prácticas desarrolladas, que siguen ancladas en modelos biomédicos, prescriptivos y centrados en la condición clínica, en detrimento de enfoques centrados en la promoción del desarrollo, el fortalecimiento de las competencias familiares y la garantía de los derechos^(19,23,35)^. Este escenario revela la urgente necesidad de procesos permanentes de educación en salud, alineados con las directrices de la PNAISC^(64)^ y los contenidos de los Manuales de Atención a la Salud de la Persona con Discapacidad^(68,69)^, que enfatizan la centralidad de la familia en el cuidado y la actuación colaborativa entre salud, educación y asistencia social.

La escasa eficacia de las acciones de vigilancia del desarrollo, sumada a la baja utilización de instrumentos como el Marco Legal de la Primera Infancia^(66)^, las Guías para la Atención Médica de Niños con Trastornos del Neurodesarrollo^(67,69)^ y los referentes de la propia PNAISC, denuncia una actuación de la APS que sigue siendo insuficiente frente a las complejas necesidades de estas familias. Esto no solo debilita la detección precoz de afecciones, sino que también amplía las desigualdades en el acceso, ya que una parte significativa de las intervenciones acaba desplazándose a los servicios de media y alta complejidad, lo que contraviene los principios organizativos de la red y sobrecarga a las familias socialmente vulnerables^(24,34,55)^.

Además, se observa una laguna significativa en la actuación de la APS con los adolescentes con discapacidad y/o trastornos del desarrollo neurológico. Los resultados de esta revisión indican que las acciones de la atención primaria tienden a concentrarse en la primera infancia, dejando de lado las especificidades de esta población en fase de transición, que requiere enfoques interdisciplinarios continuos y sensibles al ciclo de vida. A esto se suma la escasez de directrices clínicas específicas dirigidas a estos públicos en el ámbito de la APS, lo que debilita la actuación profesional, restringe la integralidad de la atención y compromete la coordinación de las acciones entre los distintos niveles del sistema de salud.

En este contexto, es imprescindible replantearse los procesos de trabajo en la APS, de modo que las prácticas se reorganicen a la luz de la atención centrada en la familia, la comunidad y el desarrollo humano. La implementación efectiva de las directrices de la PNAISC y la RCPD exige que las unidades de salud incorporen, de manera sistemática, acciones de vigilancia del desarrollo, visitas domiciliarias calificadas, elaboración de proyectos terapéuticos singulares y actuación articulada con la red intersectorial. Esto implica no solo la formación técnica, sino también cambios culturales en el interior de los servicios, capaces de deconstruir prácticas capacitistas y biomédicas, promoviendo así una APS verdaderamente inclusiva, resolutiva y promotora de la equidad^(27,44,67)^.

## CONCLUSIÓN

Esta revisión refuerza los impactos personales, familiares y sociales que un diagnóstico de discapacidad y/o trastorno del desarrollo neurológico tiene sobre las familias, haciéndolas sufrir en varios niveles, ya sea por el sentimiento de duelo anticipado que experimentan ante el diagnóstico, ya sea por el movimiento de estigmatización y exclusión aún perceptible en la sociedad.

Evidencia las repercusiones negativas de estos diagnósticos, la falta de preparación en el manejo y cuidado de estos niños/adolescentes y la [re]organización de las configuraciones familiares ocasionadas, en su mayoría, por el abandono de los cónyuges, lo que hace que las madres sean las únicas responsables de la crianza de los hijos diagnosticados. Refuerza la dificultad reportada de acceso a la APS y la dependencia que estas familias tienen de los programas y prestaciones sociales y de seguridad social.

La revisión también señala que los profesionales de la salud que trabajan en la APS tienen poca habilidad técnica para abordar a los niños/adolescentes con discapacidad y/o trastornos del desarrollo neurológico y a sus familias, lo que da lugar a acciones puntuales basadas únicamente en la condición de salud, con acciones prescriptivas y focalizadas: un modelo biomédico de asistencia dirigido exclusivamente al niño, cuando no a sus necesidades. Se evidencia la dificultad de los equipos y las unidades de salud para establecer vínculos consistentes con estas familias, además de la necesidad imperiosa de reconocerlas como unidades centrales de la atención. Es esencial la actuación proactiva de los equipos de salud, principalmente de enfermería, en el fortalecimiento de estos vínculos, a fin de favorecer el desarrollo del potencial familiar y permitir la identificación precisa de las necesidades de salud. Se observa, además, un conocimiento superficial por parte de los profesionales sobre conceptos fundamentales como la atención integrada en redes, la longitudinalidad de la atención, las líneas de atención y el uso del PTS y el VD, además del desconocimiento o la poca familiaridad con la legislación específica dirigida al apoyo de esta población.

Los resultados de esta revisión evidencian que, aunque la atención primaria de salud desempeña un papel estratégico en el cuidado de las familias de niños y adolescentes con discapacidad o trastornos del desarrollo neurológico, persisten importantes lagunas relacionadas con la formación de los equipos, la escucha cualificada de las familias y la articulación intersectorial. Superar estas limitaciones requiere el reconocimiento de estos niños como sujetos de derechos y la corresponsabilidad de los diferentes niveles de atención y sectores involucrados. Garantizar una atención continua, integral y respetuosa pasa por comprender la diversidad de experiencias familiares y construir prácticas sensibles a sus necesidades, que no se limiten a la condición clínica, sino que afirmen su dignidad y ciudadanía.

Además, pocos estudios reconocen el protagonismo de los propios niños y adolescentes con discapacidad o trastornos del desarrollo neurológico en el proceso de atención, lo que contradice el principio de participación activa defendido por el movimiento de personas con discapacidad, sintetizado en el lema “Nada sobre nosotros sin nosotros”. Esta ausencia merece ser cuestionada y abordada en los servicios de APS, en la investigación y en la formulación de políticas públicas.

## Data Availability

Todo el conjunto de datos que apoya los resultados de este estudio fue publicado en el propio artículo.
